# Social and environmental transmission spread different sets of gut microbes in wild mice

**DOI:** 10.1038/s41559-024-02381-0

**Published:** 2024-05-01

**Authors:** Aura Raulo, Paul-Christian Bürkner, Genevieve E. Finerty, Jarrah Dale, Eveliina Hanski, Holly M. English, Curt Lamberth, Josh A. Firth, Tim Coulson, Sarah C. L. Knowles

**Affiliations:** 1 Department of Biology, University of Oxford, Oxford, UK; 2 Department of Computing, University of Turku, Turku, Finland; 3 Department of Statistics, TU Dortmund University, Dortmund, Germany; 4 Department for the Ecology of Animal Societies, Max Planck Institute of Animal Behaviour, Constance, Germany; 5 School of Biology and Environmental Science, University College Dublin, Dublin, Ireland; 6 School of Biology, University of Leeds, Leeds, UK; 7 Department of Biology, University of Konstanz, Constance, Germany

**Keywords:** Microbial ecology, Animal behaviour, Bayesian inference, Community ecology

## Abstract

Gut microbes shape many aspects of organismal biology, yet how these key bacteria transmit among hosts in natural populations remains poorly understood. Recent work in mammals has emphasized either transmission through social contacts or indirect transmission through environmental contact, but the relative importance of different routes has not been directly assessed. Here we used a novel radio-frequency identification-based tracking system to collect long-term high-resolution data on social relationships, space use and microhabitat in a wild population of mice (*Apodemus sylvaticus*), while regularly characterizing their gut microbiota with 16S ribosomal RNA profiling. Through probabilistic modelling of the resulting data, we identify positive and statistically distinct signals of social and environmental transmission, captured by social networks and overlap in home ranges, respectively. Strikingly, microorganisms with distinct biological attributes drove these different transmission signals. While the social network effect on microbiota was driven by anaerobic bacteria, the effect of shared space was most influenced by aerotolerant spore-forming bacteria. These findings support the prediction that social contact is important for the transfer of microorganisms with low oxygen tolerance, while those that can tolerate oxygen or form spores may be able to transmit indirectly through the environment. Overall, these results suggest social and environmental transmission routes can spread biologically distinct members of the mammalian gut microbiota.

## Abstract (web summary)

Long-term high-resolution data on social relationships, space use and microhabitat in a wild population of mice (*Apodemus sylvaticus*), accompanied by sampling of the gut microbiota, show that distinct sets of microorganisms dominate social and environmental transmission routes of microbiota. Microorganisms with low oxygen tolerance are more reliant on social transmission.

## Main

Host-associated microbiotas, especially the diverse communities inhabiting the vertebrate gut, are increasingly recognised as key influencers of their host’s biology, affecting the development^1–3^, physiology^4,5^, behaviour^6–8^ and ultimately ecology and evolution of their host^9–12^. Many effects of the microbiota on host phenotypes depend on microbiota community composition, which can show vast multidimensional variation among individuals, populations and species, as well as strong temporal dynamics within individuals.

Although gut microbiota variation is thought to have important effects on animal fitness, our understanding of how different processes come together to shape microbiotas in natural populations remains limited. As with any ecological community, fundamental ecological processes will govern community assembly of the microbiota^13–15^. These include processes operating inside hosts, such as microbe–microbe interactions and host-induced selection^10^, but importantly also processes operating outside the host, which affect how microorganisms come to colonize hosts. Host-associated microorganisms live in an inherently patchy landscape, with hosts forming habitat islands in a sea of less suitable habitat through which they must disperse. As such, microbiotas have been conceptualized as metacommunities^16^, whereby microbial transmission from other hosts and the environment constitute potentially powerful forces shaping the composition of individual microbiotas^17^.

Gut microorganisms can colonize hosts through various routes. In mammals, transmission starts at birth by microorganisms in the birth canal and from the mother’s gut microbiota^18^, and continues throughout life as microorganisms spread through contacts with conspecifics as well as the wider ecosystem. Recent research has specifically emphasized the importance of social behaviour in the spread of gut microorganisms among animals^19^. Host-to-host transmission can occur either via direct contact during social behaviours, or indirectly through host microbial shedding to, and acquisition from, a shared environment. In humans, sharing a living space predicts sharing of gut microorganisms^20–24^, typically much more so than genetic relatedness^21,24^. The intimacy of social interaction also appears to be important, as human friends and spouses share more gut microorganisms than strangers, with the effect strongest among spouses self-reporting a physically close relationship^25^. Social group membership in other group-living mammals also predicts gut microbiota composition^26–34^ and within social groups, stronger pairwise social relationships tend to predict a higher degree of microbiota similarity^26–28^. Such effects have also been recently documented in less social species that do not form social groups. In wild wood mice, we recently showed that social networks predicted the sharing of gut microorganisms more strongly than genetic relatedness, seasonality and spatial proximity^35^.

A separate body of research has emphasized that microbiotas are shaped by contact with the broader natural environment, such as soil and food. For example, the gut and skin microbiota of children has been shown to be markedly influenced by variable physical contact with local biodiversity and natural soils^36–39^, and experimental soil exposure can change the gut microbiota of laboratory mice^40,41^. Gut and skin microorganisms have also been shown to spread between humans through their shared environment. For example, room sharing among students was associated with gradual homogenization of the microbiota among the residents and their room surfaces^42^. A recent study also found that human gut microorganisms can persist on built environment surfaces long enough to be transmitted between people^43^. Studies such as these challenge the idea of microbiotas as strict metacommunities (where the matrix between habitat patches is completely inhospitable, for example, as in oceanic island systems), as some gut-dwelling microorganisms clearly persist and sometimes grow outside as well as inside hosts^42–46^.

Despite evidence that both social interaction and environmental exposures can shape vertebrate gut microbiotas, these different transmission routes have not been studied together and directly compared. For example, although environmental exposure (the degree of contact with natural soils and local environments) has been implicated in driving healthy microbiota development and immune function in children^36–39^, the role of socially transmitted bacteria in the same processes has not been examined. The opposite bias prevails in studies of social transmission, with most studies ignoring the potential impact of environmental transmission (but see ref. ^47^). Moreover, a common confounding factor in many observational studies is shared environmental selection, where hosts living in similar habitats may share microorganisms because of exposure to similar selective forces such as diet or stress levels, causing hosts to ‘filter’ similar microorganisms from the environment. Few studies have attempted to disentangle different transmission and selection processes, leaving considerable uncertainty about their relative importance in shaping the gut microbiota.

Importantly, bacteria vary in many attributes that may affect their propensity to transmit via different routes. Traits that influence their ability to persist and grow outside the host, such as aerotolerance and spore formation, may be particularly important in this regard^48^. For example, aerotolerant microorganisms may persist and even grow outside host guts^49^. Similarly, spore formers may be able to persist long enough outside hosts to transmit indirectly through the environment^50^. In contrast, anaerobic, non-spore-forming gut bacteria may rely on intimate physical contact to pass from host to host^51^. Consistent with such ideas, spore-forming bacteria in the human gut were found to have broader geographic range, suggesting they can spread across larger distances than those less tolerant of oxygen-rich environments^21^. However, so far, no empirical work has formally tested whether gut bacterial phenotypes predict which transmission routes are most responsible for spreading them.

In this Article, we use a tractable wild mammal system to dissect how both social and environmental transmission shape gut microbiota composition, and ask which types of microbial taxa are shared via each route. Delineating separate signals of social and environmental transmission can be challenging in some species, particularly those like humans and other primates^52^ that form tight social groups where social interactions, spatial location and other factors that shape the microbiota (such as diet) are highly correlated. We therefore opted to use a semi-social model species, the wood mouse (*Apodemus sylvaticus*). These nocturnal woodland rodents inhabit small, stable home ranges and have non-modular social networks (that is, lacking aggregation into social groups) in which social relationships are only partially related to exposure to the same space^35^. This social structure makes them well suited for disentangling social and environmental transmission effects on the microbiota. Moreover, mouse home ranges cover variable microhabitats, which when characterized and controlled for in analyses can help disentangle transmission signals from effects of similar environmental selection forces on the microbiota. In this species, social transmission of microorganisms could occur through physical contact behaviours (for example, mating, huddling, grooming, licking and fighting, Supplementary Fig. 1), whereas environmental transmission could happen through contact with microorganisms present on natural surfaces, soil, food items or faeces (though coprophagy has not been documented in this species). Within a single population over a 10 month period, we used a passive tracking system based on radio-frequency identification (RFID) technology to intensively monitor home ranges, social networks and microhabitat use, while in parallel repeatedly profiling individuals’ gut microbiota from faecal samples. With the resultant data, we then dissect how sharing of gut microbial taxa among individuals varies as a function of their social association, overlap in space use and similarity in habitat, and how microbial traits (aerotolerance and spore-forming ability) predict the extent to which microorganisms drive distinct transmission signals.

## Results

### Describing the wood mouse gut microbiota

We profiled gut microbiota composition in 362 faecal samples belonging to 189 individual mice using 16S ribosomal RNA profiling. The distribution of samples among individuals and over time is shown in Supplementary Fig. 2a. After data processing, samples had a mean read depth of 48,132 (range 7,166–450,782). These belonged to 1,455 unique bacterial amplicon sequence variants (ASVs), with each sample having, on average, 180 ASVs (range 44–322). We first explored the taxonomic composition of wood mouse gut microbiota, which we found to be broadly similar to that of wood mice in other United Kingdom populations^53,54^, and dominated by bacteria belonging to the families Lachnospiraceae (37% of ASVs), Muribaculaceae, (formerly known as S24-7; 20% ASVs), Oscillospiraceae (8% ASVs) and Ruminococcaceae (4% ASVs). The most common genera were unknown genera in family Muribaculaceae, *Lactobacillus*, Lachnospiraceae NK4A136, *Ligilactobacillus* and *Limosilactobacillus* (Supplementary Fig. 2b). Using the subset of repeat-sampled mice (mice with ≥2 samples, 255 samples from 82 individuals), we found microbiota composition to be highly individualized, with individual identity explaining 52% variation in community composition (marginal permutational multivariate analysis of variance on Jaccard index, *R*^2^ = 0.52, *F* = 2.38 and *P* = 0.001). Microbiota composition also varied temporally, with sampling month explaining 5% of compositional variation in the same analysis (*R*^2^ = 0.05, *F* = 1.67 and *P* = 0.001; Supplementary Fig. 2c) consistent with previously identified seasonal variation^54^ and microbiota similarity decreasing as time between samples increased (Supplementary Fig. 2d).

### Wood mice have weakly correlated social and spatial structure

To identify different transmission pathways for microbiota, we used logger data to derive home ranges and microhabitat profiles for each individual mouse, and social networks for the population. Social networks were built for the 150 mice present in the logger data between 1 February and 28 November 2019 (Supplementary Fig. 3a). Networks were constructed using the ‘adjusted simple ratio index’ (adjusted SRI) as a measure of social association, which reflects how often two mice were observed at the same location (logger) within 12 h of each other at times they were both known to be alive and present within the study area (Methods). This type of spatiotemporal co-occurrence, while not a direct measure of social interactions, is a commonly used proxy for social association between individuals^52,55^. Individual home ranges (space-utilization distributions) were calculated from logger data and used to derive pairwise home range overlap estimates using the Bhattacharyya index^56^. To measure whether mice were exposed to similar microhabitats, we used data from a ground cover vegetation survey to calculate an index of vegetation community similarity (Bray–Curtis index) across each pair of home ranges.

**Fig. 1:**
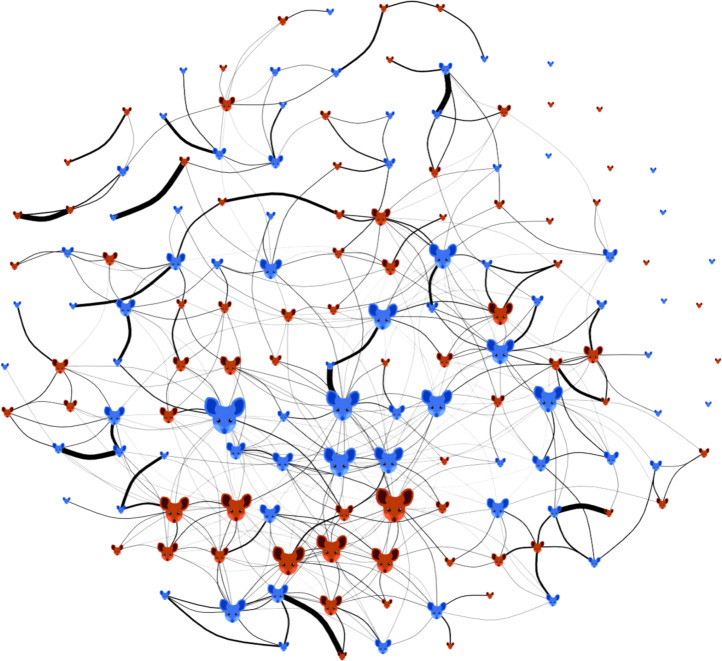
Social network of wood mice shows variation in the number and strength of social associations. The nodes are individual mice, either males (blue) or females (red). Edges are measures of social association (adjusted SRI, Methods). Node size reflects an individual’s degree, that is, the number of social connections (larger means more connections), and line thickness denotes social association strength (thicker denotes higher adjusted SRI). Nodes are arranged in a standard spring layout forced into a circular fit.

The mouse social network displayed a non-modular structure, with no clustering into social groups (Fig. 1). Across the entire 10 month monitoring period, mice had a mean of 6.4 social connections (that is, 6.4 other mice with which they occurred in the same location at least once within a 12 h window, range 0–24), though these varied considerably in association strength (mean non-zero social association index of 0.10, s.d. of 0.15 and range of 0.005–1). We also constructed separate social networks for spring and autumn (Supplementary Fig. 3b) to assess how social associations changed across the annual reproductive cycle and with the sex of pair members. In spring, the average social association strength was comparable across different sex categories (female–female, female–male and male–male pairs) but tended to be stronger in spring compared with autumn, especially in male–male pairs (Supplementary Fig. 3c). Home range overlap also varied according to season and sex; in spring, home range overlap was greatest among males, intermediate among male–female pairs and lowest among females, while during autumn, all sex categories had similar levels of range overlap (Supplementary Fig. 3c). Although habitat similarity varied widely across pairs of mice (mean Bray–Curtis habitat similarity of 0.39, s.d. of 0.16 and range of 0–0.98), it did not differ significantly between sex categories or seasons. Across the entire dataset, social association strength, spatial (home range) overlap and habitat similarity among mouse pairs were all partially correlated with each other, with the correlation between social networks and home range overlap or habitat similarity much weaker than that between home range overlap and habitat similarity (Mantel tests: social association–spatial overlap, *r* = 0.26, spatial overlap–habitat similarity *r* = 0.49 and social association–habitat similarity *r* = 0.18, all *P* < 0.001, Supplementary Fig. 3d).

### Social, spatial and habitat effects shape microbiota among mice

**Fig. 2:**
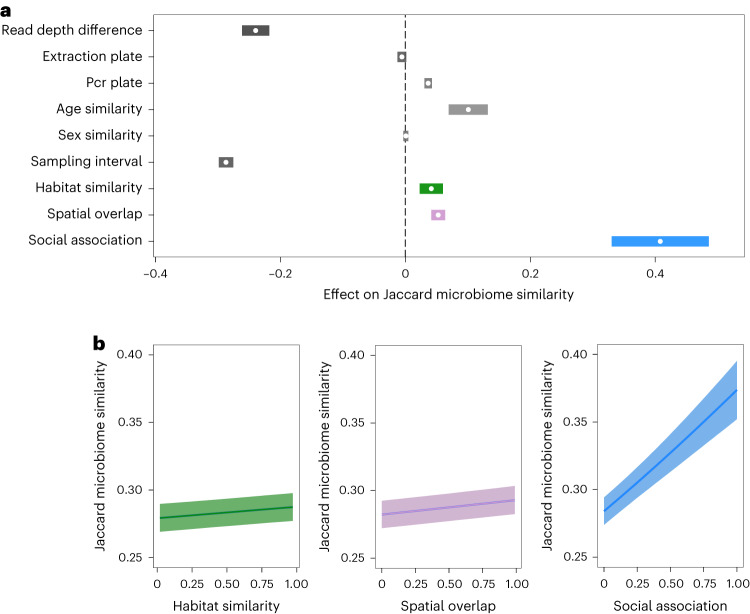
Effects of different predictors on microbiota similarity. **a**, Posterior means (points) and their 95% CIs (coloured lines) are plotted from dyadic Bayesian beta regression (Supplementary Table 1a) with pairwise microbiota similarity among hosts (Jaccard index) as the response. Posterior mean is the slope (*β*) of a given effect on microbiota similarity (for example, when the slope parameter *β* for social association (SRI) is 0.41, the model predicts a Jaccard index value of intercept +0.41 × SRI). Where CIs do not overlap zero, a variable significantly predicts microbiota similarity while controlling for all other terms shown. **b**, Slopes of the three main predictors from the same model: habitat similarity (green), spatial overlap (purple) and social association (blue), surrounded by 95% CIs (band). These slopes are conditional effects assuming mean values of other covariates.

We constructed dyadic Bayesian beta regression models (Bayesian generalized linear mixed models with family = beta) to predict the level of microbiota similarity as a function of our measures of social association, spatial overlap and habitat similarity across pairs of mice (28,855 pairwise comparisons among 241 microbiota samples from 104 individual mice). Microbiota similarity was calculated as the Jaccard index, that is the proportion of shared 16S ASVs among samples. The model results revealed that social association, spatial overlap and habitat similarity all positively predicted the proportion of microbial ASVs shared by pairs of mice, while controlling for other covariates (Fig. 2). Social association had by far the strongest effect on the sharing of gut microorganisms—over eight times stronger than the effects of spatial overlap or habitat similarity (Fig. 2 and Supplementary Table 1a). In real terms, these slopes mean that mice exposed to totally different microhabitats (Bray–Curtis habitat similarity = 0) or with completely non-overlapping home ranges are expected to share, on average, ~28% of their gut microbial taxa, while mice exposed to exactly similar microhabitats (Bray–Curtis habitat similarity = 1) or with completely overlapping home ranges would share ~29%. By contrast, mice with no social association are expected to share on average 29% of their gut microbial taxa, which increases to ~38% for mice with a social association value of 1 (always seen together).

To further explore the processes that might underpin social, spatial and habitat signals in the data, we ran similar models with two alternate response variables capturing different elements of microbiota similarity: Bray–Curtis similarity (1−Bray–Curtis distance) and the number of taxa shared by a sample pair. The number of shared taxa provides an alternative presence–absence-based measure of microbiota similarity that is unbounded (unlike the Jaccard index) and thus might be more sensitive for detecting the influence of transmission processes, which are expected to affect which taxa are shared but not necessarily similarity in their relative abundances. Bray–Curtis similarity is an abundance-weighted measure of community similarity, which is expected to respond more strongly than presence–absence-based metrics to any selective forces that drive differential within-host growth of microbial taxa. Effects were of similar magnitude in models predicting Jaccard similarity, Bray–Curtis similarity and the number of shared taxa, but social and spatial effects appeared more uncertain (displaying credible intervals (CIs) that were twice as wide) when using Bray–Curtis similarity compared with the Jaccard index or number of shared taxa (Supplementary Fig. 4 and Supplementary Table 1b,c).

**Fig. 3:**
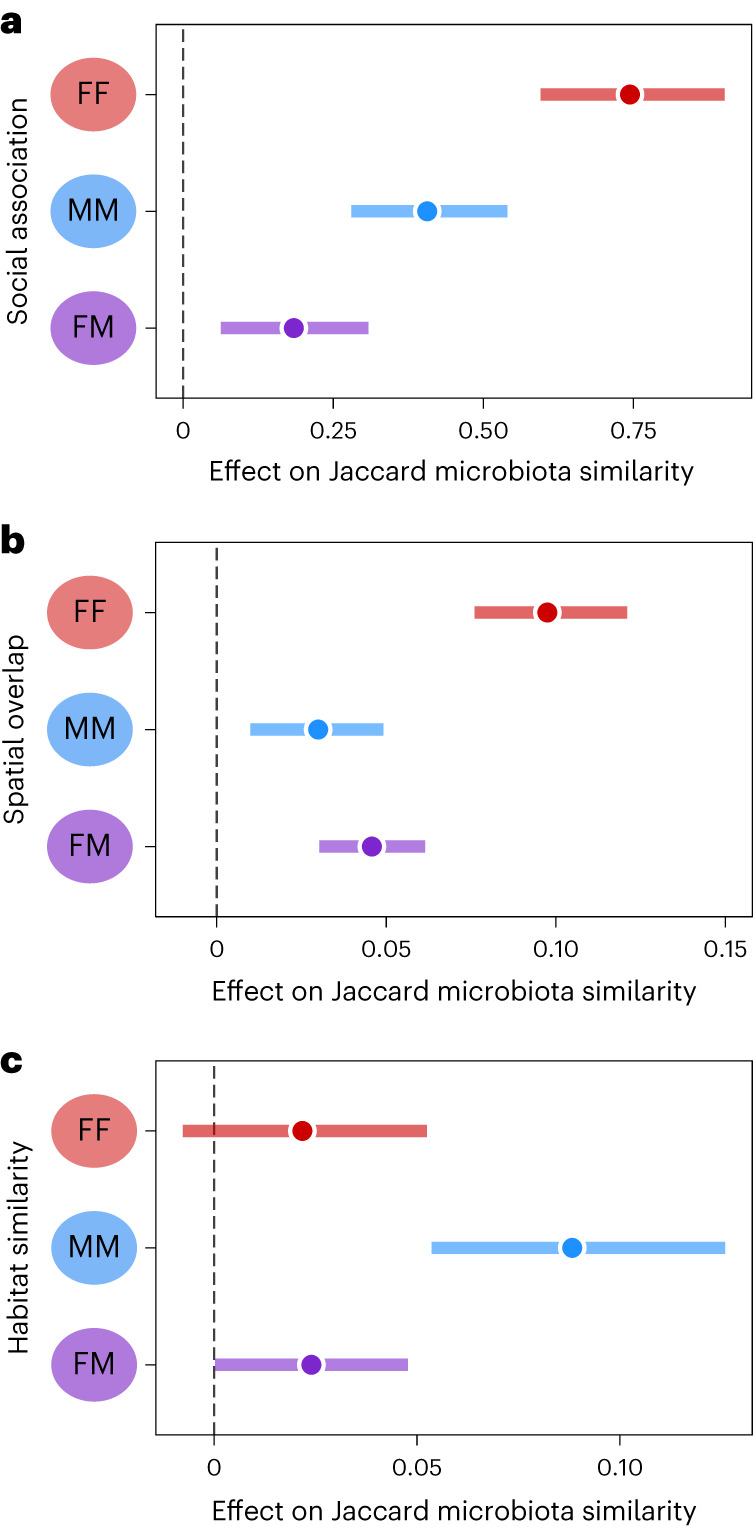
Social, spatial and habitat effects on microbiota across sex combinations. **a**–**c**, The effects of social association (**a**), spatial overlap (**b**) and habitat similarity (**c**) on Jaccard microbiota similarity (*x* axis) in pairs with different sex combinations (red is female–female (FF), blue is male–male (MM) and purple is female–male (FM)). Posterior means (points) and their 95% CIs (coloured lines) are plotted from a dyadic Bayesian beta regression (Supplementary Table 2). Where CIs do not overlap zero, the variable significantly predicts microbiota similarity.

We next explored whether the social, spatial and habitat effects on microbiota similarity varied according to the sex of individuals involved. For all three effects, we detected significant interactions with sex category (Supplementary Table 2), which showed that social association and home range overlap most strongly predicted gut microorganism sharing among female-only pairs, while the habitat effect was strongest for male–male pairs (Fig. 3). As wood mouse social behaviour may be expected to vary across breeding and non-breeding seasons, we further explored whether the sex-dependent social association effect varied between spring and autumn. This revealed that in spring (February to June) the effect of social association on gut microorganism sharing was only significant for female-only pairs, while in autumn (July to November), the social effect was driven by same-sex (both male–male and female–female) pairs (Supplementary Fig. 5 and Supplementary Table 3). Across all models, the effect of social association was strongest in female-only pairs during spring, where on the scale of the original variables it meant that while female pairs that were never observed together shared on average 30% of their pooled gut microbial taxa (ASVs), pairs with strong social associations (mice associated in over 50% of the instances they were observed) were predicted to share on average 60% of their gut microbial taxa.

### Microbial phenotypes are associated with transmission signals

We used Bergey’s Manual of Systematics of Archaea and Bacteria^57^ to classify the aerotolerance and sporulation phenotypes of bacterial genera detected in faecal samples. Since we found that home range overlap predicted gut microorganism sharing among mice (Fig. 2), we first examined which types of bacteria are detectable in both the local environment and the gut, and thus have the potential for transmission between these two environments. To do this, we profiled the soil microbiota from 25 sites across our study area using the same methods used to characterize the gut microbiota, and classified aerotolerance and spore-formation ability for bacterial genera present in the soil. Soil microbiota were more diverse than mouse gut microbiota, with 3,450 ASVs and 502 genera found in soil compared with 1,289 ASVs and 188 genera in the mouse gut (Supplementary Fig. 2). We searched for phenotype information for all genera present in mice or in both mice and soil. Among taxa found only in soil, we searched phenotype information for genera found in at least half of the soil samples. We found that while few taxa overall were shared between mouse faeces and soil, with just 6% ASVs and 24% genera detected in faecal samples also found in soil, nearly all shared taxa were aerotolerant (Supplementary Fig. 6).

**Fig. 4:**
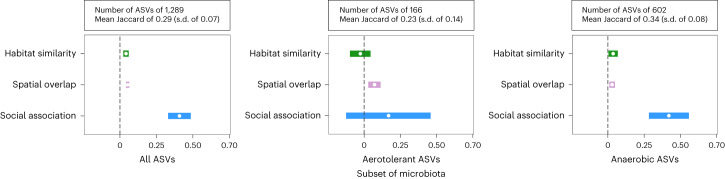
Effects of social association, spatial overlap and habitat similarity on the sharing of either anaerobic or aerotolerant gut microbial taxa. Posterior means (points) and their 95% CIs (coloured lines) are plotted from dyadic Bayesian (Supplementary Tables 1a and 5a,b) with pairwise ASV sharing among hosts (Jaccard index) as the response. Where CIs do not overlap zero, a variable significantly predicts microbiota similarity while controlling for other predictors and covariates.

Overall, we could reliably infer phenotypic information for 60% of gut microbial genera (Supplementary Table 4). Using this subset, we then used two analytical approaches to assess which kind of bacteria spread through each transmission route in this population. First, we repeated our probabilistic models using Jaccard indices calculated from one of the following four phenotypic subsets: (1) strict anaerobes (2) aerotolerant (3) spore-forming and (4) non-spore-forming taxa. As these subsets contain varying numbers of ASVs and differ in the mean proportion of taxa shared among hosts, we cannot directly compare the strength of a specific effect across models. However, we can assess how the relative strength and uncertainty of key effects within each model varies according to the subset of microorganisms being considered. When considering only aerotolerant taxa, the social network’s effect on ASV sharing became weaker and less certain compared with the effects of spatial overlap and habitat similarity, to the extent it was no longer significant (Fig. 4 and Supplementary Table 5). In contrast, when only strictly anaerobic taxa were considered, the relative magnitude of effects mirrored that observed for all taxa, with the social network predicting sharing of ASVs among mice more strongly than shared use of space or habitat similarity (Fig. 4 and Supplementary Table 5). Overall, these results suggest that anaerobic taxa predominantly drive the effect of social association observed. In contrast, subsetting microorganisms by their ability to form spores did not appreciably alter the relative magnitude of any of the effects (Supplementary Table 5 and Supplementary Fig. 7).

**Fig. 5:**
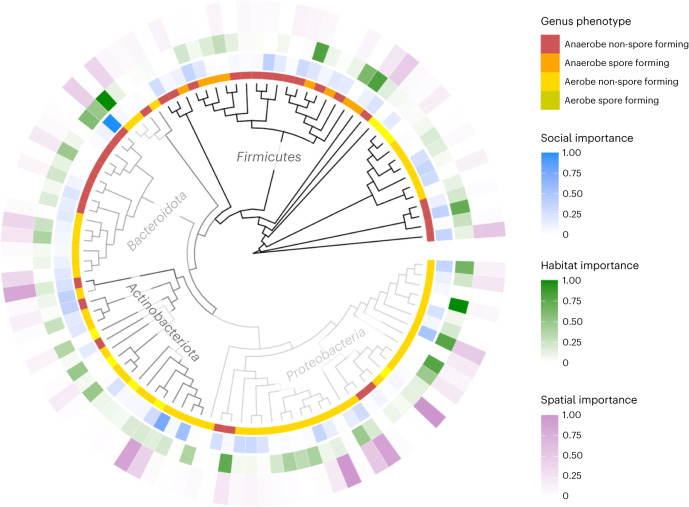
Phylogenetic distribution of bacterial phenotypes and importance values for transmission signals across gut microbial genera. The figure covers the genera that were included in the models, that is, the 110 genera with complete phenotypic information out of 188 found in the mouse gut.

**Fig. 6:**
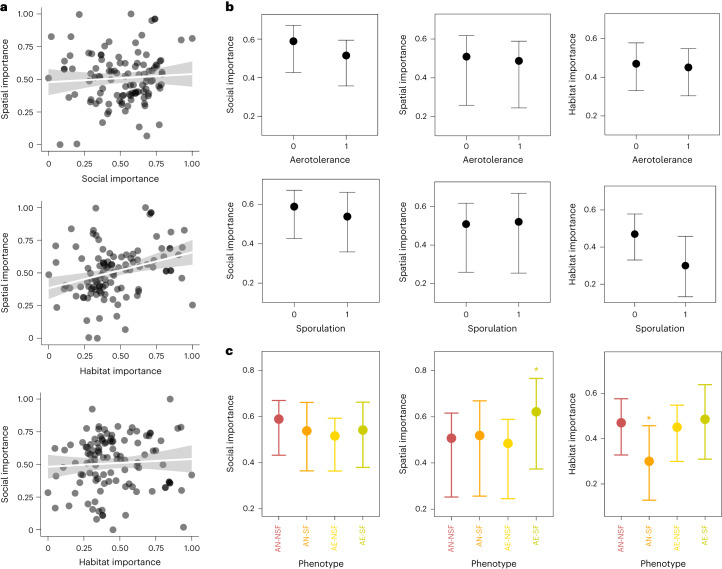
Distribution of importance scores and bacterial phenotypes. **a**, Correlations among importance scores for the social, spatial and habitat signals (line), with a 95% confidence interval (band). **b**, The statistical effects of aerotolerance or sporulation on social, spatial and habitat importance values (*y* axis). These effects are based on a phylogenetically controlled Bayesian Gaussian regression (Supplementary Table 6) predicting importance scores with aerotolerance, sporulation and their interaction across *n* = 110 unique bacterial genera. The whisker lines denote the conditional effects of phenotype on each importance score, with the point being the posterior mean and whiskers marking the 95% CIs. **c**, The statistical effects of phenotype combinations (AE-SF is aerotolerant spore former, AE-NSF is aerotolerant non-spore-former, AN-SF is anaerobic spore former and AN-NSF is anaerobic non-spore-former) on social, spatial and habitat importance values (*y* axis). This is based on a phylogenetically controlled Bayesian Gaussian regression (Supplementary Table 7), predicting importance scores with phenotype combination categories across *n* = 110 unique genera. The whisker lines denote the conditional effects of phenotype on each importance score, with the point being the posterior mean and whiskers marking the 95% CIs. Phenotypes marked with an asterix differ significantly from other phenotypes in their values of given importance (post hoc model, Supplementary Table 8).

Second, for each bacterial genus we calculated ‘importance scores’, which capture their impact on the model’s ability to detect social, spatial and habitat effects, respectively. We did this by dropping each genus in turn from the data, recalculating the Jaccard index, re-running our main model and measuring the extent to which uncertainty (CI width) increased around each effect when that genus was excluded. ‘Important’ genera for a given effect are therefore those that increase the signal-to-noise ratio. In all models where a single genus was excluded, we found social, spatial and habitat effects that were significant and similar in magnitude to those detected in the full model. This indicates that no single genus drove these effects, but rather each genus had a small contributory effect that varied in magnitude and direction among genera. For all three effects (social, spatial, and habitat), importance scores showed no strong phylogenetic clustering, suggesting taxa from across the bacterial phylogeny contribute to each effect (Fig. 5). However, spatial and habitat importance scores were significantly positively correlated (*r* = 0.35 and *P* < 0.001), suggesting some overlap in the taxa that are most important in generating these two effects (Fig. 6a). By contrast, neither spatial nor habitat importance scores correlated with social importance scores, implying different set of taxa were influenced by social versus environmental associations among mice (Fig. 6a). Indeed, most of the ten most socially important genera belonged to the phylum Firmicutes and none were from Proteobacteria, while most of the ten most important genera for both spatial and habitat signals belonged to Proteobacteria (Supplementary Fig. 8).

We next used Bayesian Gaussian regression models generalized linear mixed models with family = Gaussian) to formally test whether aerotolerance or spore-forming ability predict the importance of bacterial genera for social, spatial and habitat signals, while controlling for any phylogenetic structure. This revealed that, consistent with the earlier modelling approach, social importance was negatively associated with aerotolerance, (posterior mean of −0.07 and CI from −0.14 to −0.00, Supplementary Table 6a and Fig. 6b). Habitat importance was also negatively predicted by sporulation (posterior mean of −0.17 and CI from −0.31 to −0.02). To further explore effects of phenotype interactions, we then ran additional models predicting importance scores with phenotype as a four-level factor (aerotolerant spore formers, aerotolerant non-spore-formers, anaerobic spore formers and anaerobic non-spore-formers) and ran post hoc models to determine whether the most important phenotype had significantly different importance compared to others. This revealed that spatial importance scores were significantly higher in aerotolerant spore formers than genera with other phenotypes (Fig. 6c and Supplementary Tables 7b and 8b), while controlling for bacterial phylogenetic relatedness. Social importance was, on average, highest in anaerobic non-spore-formers but this effect was not significant (Fig. 6c and Supplementary Tables 7a and 8a). Furthermore, compared with other phenotypes, anaerobic spore formers had significantly lower importance for the habitat signal (Fig. 6c and Supplementary Tables 7c and 8c).

## Discussion

Recent research has shown that the mammalian gut microbiota can be influenced by transmission through social behaviours^19^ or environmental contacts^58^, but the relative contributions of social versus environmental transmission pathways have not been explored simultaneously. Here, we find evidence for distinct effects of environmental and social transmission in shaping the gut microbiota composition of wild mice. The microbiota of wood mice was affected by both their shared use of space and by social associations with other conspecifics, with these transmission pathways generating effects that differed both in magnitude and in the microbial taxa involved. Specifically, the social signal in wood mouse gut microbiota was over eight times stronger than effects of either spatial overlap or habitat similarity, meaning that mice who were often observed together (in the same place close in time) shared many more gut microbial taxa than those who only shared space or were exposed to similar microhabitats. Social contacts have been found to homogenize the microbiotas of interacting individuals of many social species^25–28^, but the fact that social transmission can have such a strong effect independent of shared space and even in a relatively non-social species such as the wood mouse, is striking. This might partly be because as a non-group-living species, wood mice vary greatly in the number and intensity of their social interactions across the population; if social contacts homogenize microbiota, very intense social interactions, such as those within social groups of group-living species, might result in low variation in microbiota composition, limiting the ability to correlate the degree of social contact with microbiota similarity.

These results concord well with earlier findings on microbiota transmission from another wood mouse population, where using more sparse behavioural monitoring (using only nine loggers across a similar sized area) we showed that social networks strongly predicted gut microbiota composition, independent of spatial proximity (a simple distance between each mouse’s average point location)^35^. Notably, unlike in the earlier study, the findings here are based on behavioural data collected with completely unbaited loggers recording completely natural space use behaviour, suggesting that strong social effects on the gut microbiota are general across wood mouse populations and readily detectable using different tracking methods. The finding that home range overlap and habitat similarity had generally small effects on microbiota sharing is also in line with earlier findings from other wood mouse populations where geographic location (for locations 1–6 miles apart) explained a relatively small amount of microbiota variation^53,54^.

As with all observational studies, the social, spatial and habitat effects we detect here could, in principle, be capturing a number of different processes. For instance, in some animal species, populations are spatially or socially structured according to relatedness, such that host genetics might contribute to associations between behaviour and microbiota similarity. While we did not assess relatedness in the present study, we consider this possibility unlikely, as previous work in a nearby wood mouse population showed no relationship between kinship and microbiota composition and only a weak relationship between kinship and social structure^35^. Furthermore, by examining how the social, spatial and habitat effects we identify vary according to the microbiota similarity metric used, we gain more insight to the processes generating them. Since transmission processes are expected to affect which taxa colonize a host but not necessarily how well they subsequently grow, we expected transmission signals to be more readily captured using presence–absence-based measures of microbiota similarity rather than those that factor in taxon abundances. Conversely, selective effects (where environmental conditions drive similar patterns of within-host microbial growth) are expected to be more readily detectable using abundance-weighted similarity metrics. Consistent with our interpretation that the social and spatial effects reflect transmission processes, we found that both of these effects were twice as uncertain in models where an abundance-weighted metric was used (Bray–Curtis similarity) compared with a presence–absence metric (Jaccard index). Meanwhile, the effect of habitat similarity showed more comparable uncertainty in models using binary versus abundance-weighted similarity metrics, suggesting this effect may be under some influence from environment-driven selective effects such as diet, though further work would be required to directly test this. Overall, therefore, we conclude the social and spatial effects we detect are probably driven by differential microbial transmission among hosts.

When gut microorganisms are transmitted through social contact, their distribution across the host population can reflect the social structure among hosts. Consistent with this, we found that the social network’s effect on microbiota composition varied between sexes and across seasons. The social effect on microbiota composition was strongest for female–female pairs and weakest for female–male pairs, with male–male pairs having an intermediate effect size. The social effect was particularly strong in female–female pairs during spring, when social associations were stronger on average. This seasonal difference may be linked to behavioural differences of wood mice across their breeding cycle. During the breeding season (approximately June to November in Wytham), wood mice, especially females, are more solitary and territorial compared with the non-breeding season, when multiple mice may co-nest together in same-sex groups^59–62^. Interestingly, the pattern of sex dependency in the social transmission effect detected here differed from earlier findings from another wood mouse population, where social association predicted microbiota similarity only in male–male and male–female, but not female–female pairs^35^. More detailed behavioural data would be needed to understand which specific types of social interaction are involved in gut microbial transmission, but our results suggest that the fine-scale social behaviours that spread microorganisms between mice may vary according to the population of mice as well as sex or breeding status even among individuals utilizing the same space.

Our results clearly show that not all microorganisms are equally spread by a given transmission route. Through two complimentary analytical approaches, we found that different microbial taxa underpinned social effects on the microbiota compared with effects of spatial overlap and habitat similarity, implying that different taxa transmit through social contacts and the shared environment, respectively. Specifically, our social transmission signal was driven by sharing of anaerobic bacteria, as it was no longer significant when they were removed from the analysis. Deconstructing the whole-community-level effects into genus-level contributions further revealed that spatial signal in the microbiota was driven most strongly by aerotolerant spore-forming genera. Sampling the microbiota of the environment (soil), also revealed that the subset of microorganisms present in both soil and mouse gut were almost exclusively aerobes. We do not expect these soil microorganisms to represent a full picture of microorganisms present in the mouse environment, but the fact that aerobic bacteria heavily dominated those shared between soil and mice supports the idea that aerotolerance is important for environmental transmission of gut microorganisms while anaerobic taxa may require a different mode of transmission. In future, more thorough sampling and source-tracking approaches could identify the substrates through which indirect host-to-host transmission happens.

Other evidence for a link between the aerotolerance and transmission mode of gut microorganisms exists in the literature. Studies of wild baboons found that social associations predicted microbiota similarity and this effect was driven by anaerobic and non-spore-forming bacteria^28^. A follow-up study further showed that baboon populations living in different geographic locations differed specifically in the aerobic microbiota they hosted^47^. Studies on the human gut microbiota have also suggested links between bacterial phenotypes and transmission ecology. For example, spore-forming taxa are more prevalent in the human gut microbiota than non-spore forming taxa, consistent with them more readily transmitting among hosts^50^, and microbial strains shared among people from geographically distant locations were more likely to be aerotolerant and spore forming than those shared among household members^21^. Alternative evidence comes from a laboratory rodent experiment in which microbial transmission between separately caged mice (‘horizontal transmission’) was driven by aerotolerant taxa, while obligate anaerobes were found to be only vertically transmitted (passed from mothers to offspring at birth)^63^.

Future research could usefully assess whether vertically transmitted gut bacteria overlap with those taxa naturally transmitted horizontally through intimate social contacts later in life. If so, this would mean that the same (perhaps anaerobic, non-spore-forming) microbial taxa spread through both social contacts and from mother to offspring. Consistent with this idea, our recent study on wood mice found that as young mice age, maternal transmission processes are gradually replaced by social transmission processes^64^, both driven by microorganisms from the same anaerobic non-spore-forming family, Muribaculaceae^35,64^. Over evolutionary time scales, this kind of transmission ecology might lead to microorganisms getting stuck not just inside a host social network but also their phylogeny, possibly leading to microorganisms transmitted through intimate interactions becoming more specialized in a given host species. Supporting this, the most host-specific gut microbial taxa were recently found to be enriched in anaerobic phenotypes across mammalian hosts^65^. A recent simulation study also suggested that the gut microbial transmission mode (horizontal versus vertical) could predict the level at which bacteria establish a stable host–microbe relationship across evolutionary time, with microorganisms less able to persist outside hosts evolving more a host-specific lifestyle^66^. However, like the above-mentioned laboratory mouse study^63^, this simulation study contrasted maternal ‘vertical transmission’ with general ‘horizontal transmission’, pooling together all microbial transmission processes among adult animals, thus not considering the separate effects of social and environmental pathways. Rather than contrasting vertical and horizontal transmission, future research might benefit from categorizing transmission processes into maternal, social and environmental pathways.

If aerobic and spore-forming versus anaerobic and non-spore-forming microorganisms spread from host to host through somewhat different transmission pathways, as our data suggest, this has two important implications. First, anaerobic non-spore-forming microorganisms that require more intimate transmission routes may be more likely to evolve a more stable relationship with their host (as suggested by Leftwich et al.^66^) and perhaps more mutualistic relationship as well, if their fitness depends heavily on one host species (as suggested by Moeller et al.^63^ on the basis of Brown et al.^67^). Compared with environmental transmission, social transmission may therefore be expected to spread microorganisms with greater functional significance for the host, for example, in nutrition^68^ or protection against pathogenic infections, as has been shown in some insect systems^69^. Second, if some microorganisms only live in and transmit between hosts while others readily spread between the host and the external environment, this calls into question the relevance of viewing host-associated microbiotas as strict metacommunities like island ecosystems. While some assumptions in classic metacommunity ecology (such as the existence of a completely uninhabitable matrix separating habitat patches) have been updated to better model the microbiota^46^, assuming that all microbiota members can similarly persist in the environment also seems unrealistic. Microbial taxa may vary greatly in the extent to which they experience the host as a true island. For instance, anaerobic microorganisms may well live in a strict metacommunity, structured by the host social network, while aerotolerant microorganisms may experience a much more continuous landscape, more analogous to valleys amid hills than islands in the sea.

Going forward, to better understand the transmission ecology of different microbiota members we will need more work to characterize microbial phenotypes that affect persistence outside the host. Data on aerotolerance and spore formation is still lacking for many animal gut microbial genera. Furthermore, aerotolerance and spore formation are probably not the only relevant phenotypes determining gut microbial transmission pathways. For instance, environmental transmissibility could be affected by persistent states mediated by toxin–antitoxin systems, low metabolism ‘viable but non-culturable’ states or morphological adaptations in the cell wall^50^. In fact, a recent thorough exploration of the human gut microbiota transmission landscape found that cell wall properties (as described by a Gram stain) were associated with human-to-human transmissibility of gut microorganisms^21^. As culture-based phenotypic information can be limited, especially for microorganisms in wild host species, the growing number of tools developed for predicting bacterial phenotypes from genomic data (for example, Traitar^70^) hold much promise. Here, a potentially useful but so far unexplored method for classifying aerotolerance phenotypes from bacterial sequence data could be to characterize ribonucleotide reductase enzyme genes^71^.

Our findings on the gut microbiota’s transmission ecology, such as anaerobes requiring more intimate contact to spread, may well generalize to other species, including humans. This highlights the need for further research on the transmission dynamics of not only pathogens but also commensal members of the human microbiota. Humans are a socially flexible species capable of large-scale modification of their own social contact network. Reducing social contact is an effective way to reduce pathogen spread, but multiple studies have also highlighted that we know essentially nothing about the consequences of social isolation for our commensal microbiota and microbiota-mediated health^19,51,72^. At the same time, a growing body of evidence emphasizes how diminishing contacts with the natural environment among urbanized human populations can have negative health consequences through a lack of natural microbiota transmission from biotic environments and consequent disruptions to immune development^36–39,73^. If isolation from natural sources of environmental microbiota transmission can compromise host health, what might be the consequence of isolating from natural sources of social transmission? Notably, both reduced environmental contact and reduced social contact are common aspects of urban lifestyles. These forms of isolation may independently disrupt the transmission networks of human microbiota^51^, reflected by the observations that urban lifestyles are linked with a range of immune disorders^74,75^ and seem to be depleting the diversity of human gut microbiota^76,77^.

## Methods

### Field data collection

During February to November 2019, we collected faecal samples and tracked the movements of 164 wild wood mice living within a woodland plot (Holly Hill) in Wytham Woods, Oxford, United Kingdom (51.77° N, −1.33° W). This involved fortnightly trapping to tag mice and collect samples, alongside continuous passive tracking of tagged individuals using RFID technology. The focal study area, where mouse behaviour was tracked, was a 2.56 ha (160 m × 160 m) ‘core grid’ but to minimize edge effects, mice were trapped and tagged from an area larger than this core grid, from a 4 ha (200 m × 200 m) ‘extended grid’ spanning up to 40 m outside of the core. Trapping sessions were carried out in the area from November 2018 to November 2019, with captured mice aged and sexed, and injected with a subcutaneous passive integrated transponder (PIT) tag for permanent identification and tracking. After processing, all individuals were released as soon as possible on the same day at the exact location they were trapped. Faecal samples for microbiota analysis were collected from the traps of identified individuals into sterile sample tubes with sterile tweezers and frozen at −80 °C within 4 h of collection. All traps showing signs of rodent presence were carefully washed and sterilized in bleach solution before the next trapping session to eliminate cross-contamination. Additionally, at the beginning of the study period (between November 2018 and February 2019), 25 soil samples were collected from around the 4 ha extended grid to serve as a general reference for the local soil microbiota. A soil sample was collected by digging a spoonful of soil (∼200 mg) from 3 cm underground, creating a mix from three digging spots within a metre of a mouse trapping location.

Mouse behaviour was monitored with a set of custom-built RFID loggers distributed across the study site, recording the time-stamped presence of any individual (PIT tag) that came within its read range (∼1 m^2^). Loggers were all unbaited and comprised 60 ‘above-ground’ loggers and 60 ‘burrow loggers’ (used for home range analysis only). Above-ground loggers were positioned evenly across the grid and rotated such that each 10 × 10 m grid cell of the study site was covered by a logger for a fortnight every 2 months, that is, 25% of the time. Burrow loggers were positioned for the autumn period (July to November) on mouse burrows approximately evenly across the grid. Further details of logger devices and the tracking protocol can be found in Supplementary Fig. 9.

Shortly after the study (May 2020), we completed a thorough survey of vegetation and microhabitat variation across the study site, in which the percentage cover by each of the eight main ground cover types in the area was recorded for each 10 × 10 m grid cell of the plot (Supplementary Fig. 10).

### Social network construction

Social networks were constructed using data from all 60 above-ground loggers for the full 10 month period (‘full social network’) as well as separately for spring (February to June) and autumn (July to November) (‘seasonal networks’). This division of the year into ‘seasons’ was done based on cutting the whole study period in two equal halves, but it also approximately mirrors the natural seasons of wood mouse breeding. Here, juveniles started appearing in the data only in early September and stopped appearing at the start of December, meaning that females were typically pregnant from August onwards. While reproductively active, female wood mice are less social and more territorial^61,62,78^. Networks were constructed and visualized using our custom social network inference and plotting functions in R^79^ with the help of R package igraph^80^. These functions took the logger data, consisting of time-stamped observations of tagged individuals in fixed locations, and calculated a pairwise association index for all mouse pairs based on the frequency with which they were observed at the same location during the same short time window. While spatiotemporal co-occurrence is not a direct measure of social interactions, it is a commonly used proxy for social association between individuals^52,55,81,82^. Logger data were first filtered to include only the normal hours of activity for this nocturnal species (16:00–08:00) and to consider detections within a unique minute. For each of these logging ‘nights’, a pair of mice were considered ‘associated’ if they were observed at the same location within 12 h of each other, consistent with our previous work^35^. These instances of association were then used to calculate an association index defined as:

$${\mathrm{Adjusted}}\,{\mathrm{SRI}}=\frac{X}{\left[X+{y}_{{AB}}+{yA}+{yB}\right]}$$

where *X* is the number of nights in which individuals *A* and *B* were observed associated (in the same location within 12 h of each other), *y*_*AB*_ is the number of nights in which *A* and *B* were both observed but not associated (observed at the same location but more than 12 h apart) and *y*_*A*_ and *y*_*B*_ are the number of nights in which both were known to be alive but only *A* or *B* was observed, respectively. Accounting for lifespan overlap in this metric allows us to more accurately summarize the temporally fluctuating social structure of the mouse population in one static social network.

### Estimating home range overlap

An animals’ home range is commonly presented as a utilization distribution describing the probability of space use with respect to time^83^. We quantified home ranges from logger data using an autocorrelated kernel density estimator^84^, implemented using the ctmm package^85^. Home range boundaries were delineated at the 75% level to provide an estimate of the core home range, the smallest area that one could expect to find a given individual inside with 75% probability. Each individual mouse’s home range was described as a three-dimensional probability distribution of space utilization, where the two base dimensions were actual space and the third dimension was utilization intensity, that is, how frequently the mouse used a given region within its range. Home ranges were calculated only for 104 (out of the total of 157) individuals satisfying our criteria for a complete and stable observation record, based on variograms estimating temporal autocorrelation in spatial records (Supplementary Appendix 1). Among these mice, we calculated home range overlap for each mouse pair using the ‘overlap’ function in ctmm utilizing the Bhattacharyya coefficient^56^. Since reliable home range estimation requires a considerable amount of tracking data, home ranges were estimated using all available logger data from both above-ground and burrow loggers. More details on home range analysis are given in Supplementary Appendix 1.

### Estimating habitat similarity

Habitat similarity between mice was estimated using data on the percentage cover by each of the eight main ground cover types within each mouse’s home range (75% core kernel density area). The main ground cover types were defined as (1) open ground (no plant coverage), (2) dog’s mercury (covered by *Mercurialis perennis*), (3) bluebell (covered by *Hyacinthoides non-scripta*), (4) bramble (covered by *Rubus fruticosus*), (5) grass (covered by grass species in the family *Poaceae*), (6) sedge (covered by *Carex pendula*), (7) Enchanter’s nightshade (covered by *Circaea lutetiana*), (8) wild garlic (covered in *Allium ursinum*) and (9) currant (covered by *Ribes spicatum*) (Supplementary Fig. 10). For each mouse, we calculated normalized abundance for each ground cover type, as the sum of its coverage across the home range (in m^2^) divided by the home range area. Using these values, we then used package ‘vegan’ to calculate habitat similarity for all pairs of mice, using the Bray–Curtis index^86^.

### Microbiota profiling

We profiled microbial communities by extracting DNA from faecal and soil samples and using primers 515F and 926R (ref. ^87^) to amplify and sequence the V4–V5 region of the 16S rRNA gene in bacteria/archaea. Full details of the laboratory work, library preparation and sequence data bioinformatics can be found in Supplementary Appendix 2. In brief, we used the DADA2 algorithm to infer ASVs from the sequence data and assigned taxonomy using the SILVA database (version 138), after which the data were decontaminated^88^ and filtered to remove non-gut microbial taxa and samples with low read counts. Finally, abundance data were normalized to the proportions of each ASV per sample.

### Statistical analyses

#### Describing microbiota variation

To characterize variation in the microbiota composition among individuals (beta diversity), we used the Jaccard index, which captures the proportion of microbial ASVs detected across a pair of individuals that are shared between them. This metric provides an intuitive way to capture transmission signals, as transmission should affect the presence/absence but not necessarily the relative abundance of taxa within a host, and our previous work suggested this metric is superior to abundance-weighted beta-diversity metrics for detecting microbiota transmission signals^35^. To assess the sensitivity of our findings to the choice of beta-diversity metric, we also modelled alternative measures of microbiota sharing: Bray–Curtis similarity and the raw count of shared ASVs between a pair (scaled between 0 and 1).

To estimate the amount of gut microbiota variation accounted for by stable differences between host individuals versus temporal fluctuations, we used principal co-ordinates analysis and marginal permutational multivariate analysis of variances (implemented with the adonis2 function of package ‘vegan’^89^) to predict the Jaccard index across samples from repeatedly sampled individuals (*n* = 255 samples from 82 individuals with a mean 3.1 samples per individual, range 2–10), using host ID and sampling month (as a factor) as fixed effects.

#### Modelling the effect of different transmission pathways on microbiota

To test the effects of social and environmental transmission on microbiota composition, we constructed a model in which microbiota similarity among pairs of mouse samples is predicted by their social association strength, spatial (home range) overlap and habitat similarity. Here, the effect of social association, while controlling for the effects of spatial overlap and habitat similarity, captures the effect of transmission via social interaction on microbiota. This is similar in logic to the so-called generalized affiliation indices, which measure intimate social affiliations as the residuals after regressing an association index on structural predictors of association, such as spatial proximity^90^. Similarly, the effect of spatial overlap controlled against the other two main predictors is meant to capture the effect of microbial transmission from and through shared space, and the effect of habitat similarity controlled against the other two predictors is intended to capture effects of both convergent exposure to similar environmental pools of microorganisms (those predicted by similar vegetation) and similar environment-driven selective forces shaping the microbiota, such as diet and stressors. To build this model, we constructed model data for a total of 28,855 dyads (all pairwise comparisons, excluding self-comparisons, among 241 unique microbiota samples from the 104 individual mice with complete home range and social network information) and used a dyadic Bayesian generalized linear mixed model implemented in package ‘brms’^91^, as validated and described in ref. ^35^ (see also ref. ^92^). This model framework allows ‘multi-membership’ random effect structures that can account for the types of dependence inherent to pairwise comparisons as well as repeated sampling of the same individuals^93^. Models used dyadic measures of microbiota similarity (Jaccard index, Bray–Curtis similarity or number of shared taxa) as the response variable, including all sample pairs except those from the same individual mouse. These values were modelled as a function of the predictor variables described above, together with a set of technical and biological covariates: host age class similarity (same versus different), sex similarity (same versus different), time interval in days between samples, sample extraction distance (the physical distance between two samples on plates during DNA extraction, as described in Supplementary Appendix 2), read depth difference and PCR plate similarity. Models with the Jaccard index of Bray–Curtis as the response used beta regression (likelihood family of beta) since the response was a proportion. Where the number of shared taxa was the response, a poisson regression was used (likelihood family of poisson). The models used default (uninformative) priors and included a multi-membership random effect (random intercept) for the identity of samples (sample A + sample B) as well as individuals (individual A + individual B) involved in each pairwise comparison. In addition to this primary model, we ran one additional pair of models to explore seasonal and sex-specific differences in social and environmental influences on the microbiota. Here, we modelled Jaccard index as a function of the same set of predictors but for each of the seasonal data subsets of the social network (spring or autumn), and included an interaction term between sex combination (three-level factor: female–female, female–male and male–male) and social association. These Bayesian models uses a Markov chain Monte Carlo sampler (Hamiltonian Monte Carlo sampler, implemented with RStan^94^ a wrapper for Stan^95^, to estimate posterior distributions^93^. We ran the models with four parallel chains, each with 1,000 warm-up samples preceding 4,000 actual iterations and used posterior checks to ensure reliable model performance^96^. Specifically, we ensured that the chains converged, Rhat values were <1.05, bulk effective sample sizes were no smaller than 10% total posterior draws and the sampler took small enough steps (adapt_delta of 0.98 and max_treedepth of 13) to avoid excess (>10) divergent transitions after warm-up.

#### Transmission signals and microbial phenotypes

We used Bergey’s Manual of Systematics of Archaea and Bacteria^57^ to classify the aerotolerance (aerotolerant or strictly anaerobic) and sporulation (spore forming or non-spore forming) of each bacterial genus identified. When this information could not be found in Bergey’s Manual (for example, as for some newly described or renamed taxa), we sought it from original research papers describing the genus or specifically assessing aerotolerance or sporulation of that bacterial genera. Full phenotypic trait data for each genus with references are presented in Supplementary Table 4. In our analyses including bacterial phenotypes, we only included ASVs belonging to the 110 genera (out of 188 genera in the full dataset) where aerotolerance and sporulation were both well known. For unknown genera in a given family, we only included in the analyses if there was substantial evidence that all members of that family were of a given phenotype. All genera with unknown, uncertain or variable phenotypes in terms of either aerotolerance of sporulation were excluded from the analysis. We used these data in two analyses to examine how different transmission signals (social, spatial and habitat effects) were related to microbial phenotypes. First, we calculated additional Jaccard indices that reflected the proportion of shared ASVs among those belonging to the following four phenotypic subsets: (1) strictly anaerobic (2) aerotolerant, (3) spore forming and (4) non-spore forming. These Jaccard indices were then used as response variables in beta regression models with social, spatial and habitat predictors together with covariates, as described above. The different phenotypic subsets of microbiota contained varying numbers of ASVs and differed in their mean similarity across hosts (Fig. 4 and Supplementary Fig. 7). Thus, we cannot directly compare the effects between models predicting these different versions of Jaccard, because they come from datasets with inherently different uncertainty, intercepts and slopes. However, we can assess how the relative strength and certainty of key effects within each model varies across models. As these phenotype-specific Jaccard values also included a few zeros (no taxa shared between two samples), to meet beta-regression criteria, Jaccard values were scaled by (Jaccard × (*n* − 1) + 0.5)/*n*, where *n* is the sample size.

Second, we quantified the importance of each bacterial genus in driving each transmission signal and then asked whether microbial phenotypes predicted variation among genera in this importance. ‘Importance’ scores for each of the 188 bacterial genera were calculated by dropping each in turn from the microbiota data, recalculating the full Jaccard Index and rerunning the above-described beta regression model^92^. The ‘importance score’ for each effect of interest (social association, spatial overlap and habitat similarity) reflected the extent to which dropping a genus from the analysis reduced the certainty of that effect estimate. Specifically, the importance of genus *G* for effect *E* was calculated as the increase in the 95% CI width (CI*w*) when *G* is excluded (CI*w*_excl_ − CI*w*_incl_) relative to the baseline CI width when *G* is included (CI*w*_incl_), divided by the square root of the number of ASVs (*n*ASV) assigned to genus *G*:

$${\mathrm{Importance}}_{{GE}}=\frac{({\mathrm{CI}}_{\mathrm{excl}}-{\mathrm{CI}}_{\mathrm{incl}})/{\mathrm{CI}}_{\mathrm{incl}}}{\sqrt{n{\mathrm{ASV}}}}.$$

The resulting values were approximately normally distributed, and were scaled between 0 and 1 to create importance scores that were on the same scale as other binary predictors in each model. Across the 110 genera with reliable phenotypic information, we tested whether aerotolerance (0/1) or sporulation ability (0/1) predicted importance scores for each effect of interest (social, spatial or habitat), using a Bayesian generalised linear mixed model (Gaussian regression) again implemented with R package ‘brms’. To control for phylogenetic non-independence among genera in these phenotypes, we also ran the model including the phylogeny among genera (in the form of a variance–covariance matrix) as a random structure in the model.

### Reporting summary

Further information on research design is available in the Nature Portfolio Reporting Summary linked to this article.

### Supplementary information

Supplementary Information Supplementary Appendices 1 and 2, Figs. 1–10, table legends 1–8 and Tables 1–3 and 5–8. Table 4 is given as a separate csv file, but its legend is added here as ‘Table 4’ and referred as such in the text.

Reporting Summary

Supplementary Table 4 Aerotolerance and spore-forming ability of bacterial genera. See full legend in the Supplementary Information pdf file, Table 4.

## Data Availability

All data used in this study are publicly available through the NERC Environmental Information Data Centre^97^.

## Code availability

R code tutorials on the social network analysis and Bayesian regression methods used in this study are available in Aura Raulo’s github (social network analysis using lifespan-corrected sdjusted simple ratio index^79^ and Bayesian regression models and drop models for construction of importance scores)^92^.
