# Supplementary material for: Social and environmental transmission spread different sets of gut microbes in wild mice
Source: Nat Ecol Evol. 2024 May 1;8(5):972–85. doi: 10.1038/s41559-024-02381-0 (PMC11090834; doi:10.1038/s41559-024-02381-0)
Supplement: Supplementary file 1 — Supplementary Appendices 1 and 2, Figs. 1–10, table legends 1–8 and Tables 1–3 and 5–8. Table 4 is given as a separate csv file, but its legend is added here as ‘Table 4’ and referred as such in the text. [file 41559_2024_2381_MOESM1_ESM.pdf]

nature ecology & evolution

Article

https://doi.org/10.1038/s41559-024-02381-0

Social and environmental transmission spread different sets of gut microbes in wild mice

In the format provided by the authors and unedited

Index

Supplementary Appendices

Appendix 1. Details on home range estimation

Appendix 2. Details on gut microbiota analysis

Supplementary Figures

Supplementary Figure 1. Examples of social and environmental contact behaviours in wood mice

Supplementary Figure 2. Composition and variation in wood mouse gut microbiota and the local soil microbiota.

Supplementary Figure 3. Variation and co-variation among social association, spatial overlap and habitat similarity.

Supplementary Figure 4. Effects on alternative measures of microbiota similarity.

Supplementary Figure 5. Effects of social association on microbiota similarity across seasons and sexes

Supplementary Figure 6. Phenotype distribution among bacterial genera found in mouse gut, soil or both

Supplementary Figure 7. Effects of main predictors on microbiota similarity in different phenotypic subsets of the microbiota – spore-formation.

Supplementary Figure 8. Overview of the top-ten most important genera for social, spatial and habitat signal in microbiota similarity.

Supplementary Figure 9: The RFID logger device and tracking protocol

Supplementary Figure 10: Main ground cover habitat types in Holly Hill study site.

Supplementary Tables

Supplementary Table 1. Model results: Effects of Social association, spatial overlap and habitat similarity on microbiota similarity.

Supplementary Table 2. Model results: Effects of social association, spatial overlap and habitat similarity on microbiota similarity am2ng pairs with different sex categories.

Supplementary Table 3. Model results: Effects of Spring and Fall social association on microbiota similarity among pairs with different sex categories.

Supplementary Table 4. Aerotolerance and spore-forming ability of bacterial genera together with their importance scores in models where excluded.

Supplementary Table 5. Model results: Effects social association on Jaccard similarity based on aerotolerant vs. anaerobic taxa.

Supplementary Table 6. Model results: Effects of phenotypes on importance scores across bacterial genera.

Supplementary Table 7. Model results: Effects of microbial combination phenotypes on importance scores across bacterial genera.

Supplementary Table 8. Model results: Results of post hoc models testing whether specific phenotype categories differ significantly from other genera in their importance.

Appendix 1. Details on home range estimation

For ease, figures mentioned in this appendix are included within this appendix and referred to as Appendix 1 – Figure A, Appendix 1 – Figure B and Appendix 1 – Figure C.

Home ranges were inferred from the logger data aggregated within ten-minute periods, such that all records of an individual at the same location within each ten minute-period were considered a single unique "logger visit". To ensure that data quality was suitable for home range analysis we first filtered out all individuals that did not have at least five unique "logger visits" in at least three unique locations. We then inspected the remaining data using empirical variograms, a method visualising the autocorrelation structure in locational timeseries by presenting the semi-variance (proportional to home range size) as a function of increasing time lags, with clear asymptotes diagnostic of range-residency (Fleming et al., 2015, See Appendix 1 -Figure A). Here, a lack of asymptote indicates either the data is too incomplete to reveal the home range extent, or that the animal is not exhibiting site fidelity (Calabrese et al., 2016). Based on this, we excluded individuals who's variogram showed a positive linear relationship between estimated distance and time lag between logger observations, as these were considered signs of either logger histories that were too incomplete for reliable home range estimation (e.g., due to small amount of data or edge effects on the study grid) or spatially shifting home range location (unstable home range). Under these criteria, home ranges could be estimated for 104 of the 157 mice recorded on loggers.

To estimate home range, for each individual we first fit a series of range-resident movement models using model selection to identify the best model fit, given the data (Fleming et al., 2015) following the workflow described by (Calabrese et al., 2016). We applied the AKDE to these movement models using an error-informed model (with error set to be 1 meter, approximately the detection diameter of loggers). The method accounts for error by first Kriging data to reduce location error before placing the kernels (Fleming et al., 2018; Fleming & Calabrese, 2017). Although the observation model of fixed station data is different to tracking data that the ADKE was developed for, the scales of movement observed for mice was larger than the grid resolution. Accordingly, visual inspection of the kernels against the grid setup and tracking data showed little evidence of excess density at the stations. Among these mice, we calculated home range overlap for each mouse pair using the "overlap" function in *ctmm* and the Bhattacaryya coefficient, defined as:

$$BC(h, g) = \int \sqrt{h(x)g(x)}\ dx$$

, where h and g are two probability distributions of value x respectively.

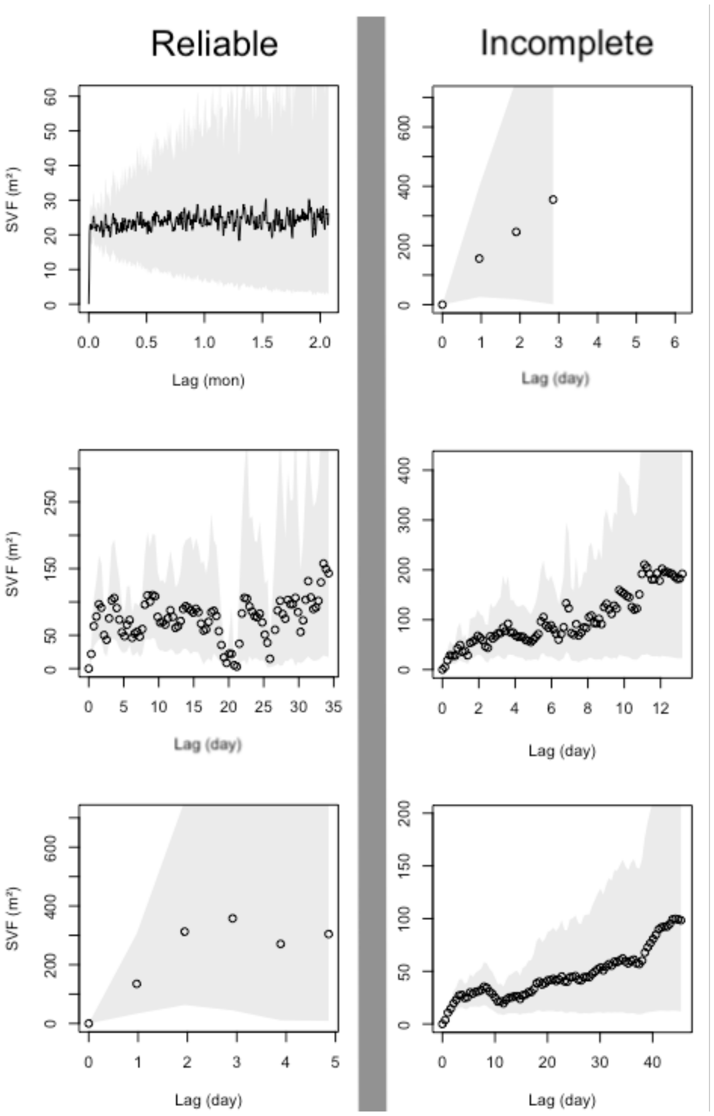

Appendix 1 – Figure A. Examples of variograms used as an inclusion criterion for home range estimation. Six example variograms describing the completeness and stability of location-observation record of six individuals. Variograms were used to filter out individuals with insufficient logger data for reliable home range estimation. Here, each box is the observation record of one individual mouse, where x-axis describes time lag in days/months between logger observations and y-axis (SVF) describes the expected distance between observations. Where the distance estimate grows linearly with time-lag between observations (right-hand panel), individual's home range is unstable (shifting) or observation data is not complete enough to capture the true home range and thus estimates for these individuals are not reliable.

Validation of using above-ground and burrow logger data with varying density for home range analysis.

As movement-model based home range analysis requires an extensive number of relocation data

points for an individual, we included observations from all logger data, from both above-ground loggers and burrow loggers, for home range analysis to maximize the number of individuals for whom home range could be reliably estimated. This induced a sampling inbalance in our data, as we had more loggers out covering the same area during Fall (n= 60 evenly spaced above-ground loggers, July-November) than in Spring (n= 60 evenly spaced above-ground loggers and 60 burrow-loggers, February-June). To ensure this was not biasing our home range estimates between seasons, we chose a subset of well-tracked mice from the fall period and created a comparable pair of home ranges for each individual using 1) the full logger data (dense data home ranges) and 2) subset of logger data from the 60 above ground loggers only (sparse data home ranges). Across example mice, these two home range estimates were largely overlapping (See Appendix 1 - Figure B below) and had area estimates that were highly correlated (r=0.97, p<0.001; Appendix 1 - Figure C below). This implies that our initial criteria for data density acceptable for reliable home range estimation were realistic, estimates were asymptotic and thus including any additional observation data had negligible influence on the estimates. Based on this, we decided to use all available logger data to infer home ranges for as many mice as possible.

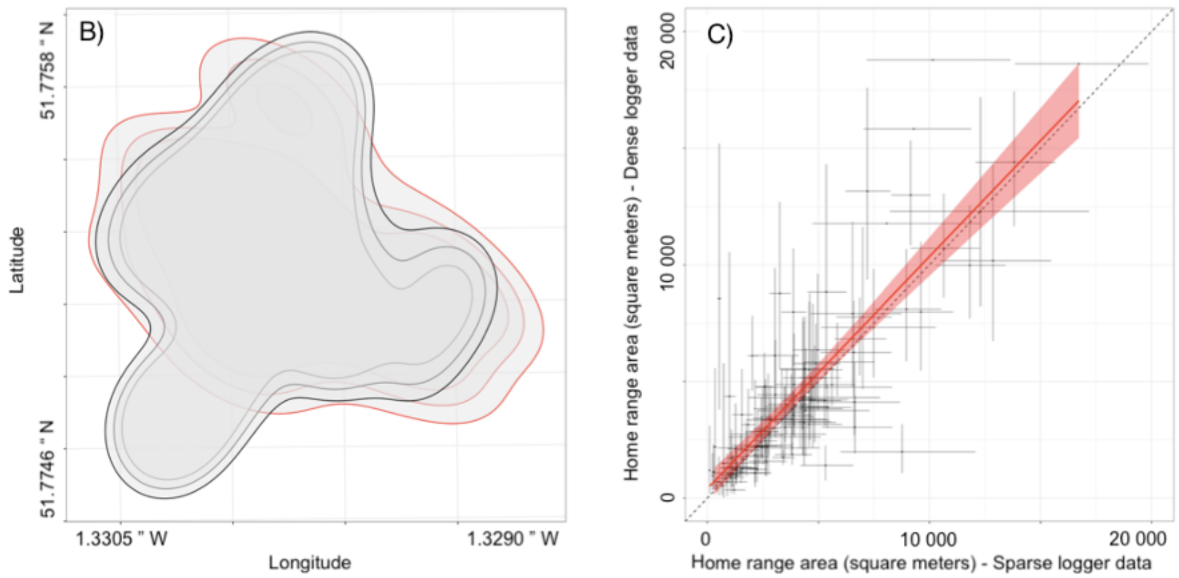

Appendix 1 – Figure B. Example of overlap between the home range of a single mouse constructed from sparser (red) vs denser (grey) logger data.

Appendix 1 – Figure C. Correlation between home range area estimates based on sparser (x-axis) and denser (y-axis) logger data, and the regression line (red line with pink band marking 95%-confidence interval) from a linear model predicting one with the other. The area estimate combinations (points) have both horizontal and vertical 95%-confidence intervals (black lines), as per the *akde* model used to estimate the home ranges.

Appendix 2. Details on gut microbiota analysis

For ease, the figure mentioned in this appendix is included in the end of this appendix and referred to as Appendix 2 - Figure A.

Sample analysis order

The microbiota samples were analysed in two batches, each including i) DNA extraction, ii) library preparation and iii) sequencing on Illumina MiSeq platform. Both batches included soil samples as well as faecal samples of two rodent species (Wood mouse, *Apodemus sylvaticus;* Yellow-necked mouse*, Apodemus flacvicollis*). The first batch had additionally faecal samples from bank voles (*Myodes glaerolus*). All samples were randomised across 96-well plates for DNA extraction and re-ordered for 96-well plates for library preparation to enable detection of extraction and PCR-related technical effects on the final data. Sequencing batches had a slight temporal difference: First batch contained only samples from the first half of the study (February-July), while the second batch contained samples from throughout the study (February-November).

DNA extraction

DNA was extracted from samples with a 96-well plate format Zymo Quick-DNATM Fecal/Soil Microbe kits (Zymo) according to manufacturer's instructions, using a Qiagen TissueLyser plate-format bead beater for DNA homogenization. Each DNA extraction plate contained one mock community (ZYMOBiomics Microbial Community Standard cat. No.D6300) to evaluate extraction, PCR and sequencing accuracy. After extraction, DNA concentrations in samples and controls were quantified with a Qubit 3.0 Fluorometer to verify extraction success. Control samples were additionally amplified for bacterial DNA with a 40-cycle PCR (see below) and product run on gel to make sure no bacterial DNA amplified from negative controls. No bands were seen on these gels.

Library preparation and sequencing

Extracted DNA samples were re-ordered for library preparation plates and amplified in a two-step PCR with primers 515F and 926R targeting an approximately 370 bp sequence in the V4-V5 region of the bacterial 16S rRNA gene (Walters et al., 2016). Specifically, primers were as follows:
Forward primer:
AATGATACGGCGACCACCGAGATCTACAC_INDEX_ACACTCTTTCCCTACACGACGCTC
, where "INDEX" denotes a variable forward index from the set of Illumina Nextera barcoded indices, which we used in a dual-indexing protocol to mark each of the 96 samples on a plate with a unique combination of one of the 8 forward and one of the 12 reverse indices. The region before the index is the forward adapter region to match flow cell surface. The region after the index is forward region to match the overhang from the first round pcr.

Forward primer:
CAAGCAGAAGACGGCATACGAGAT_INDEX_ GTGACTGGAGTTCAGACGTGTGCTC
, where “INDEX” denotes a variable reverse index, the region before the index is the reverse adapter region to match flow cell surface and the region after the index is the reverse region to match the overhang from the first round pcr.

Each plate contained 95 samples and one negative PCR-control. First round PCR had 20µl reaction volume, consisting of: 10µl KAPA 2x Mastermix (KAPA Biosystems), 0.25µl each primer at 10µM, 4.5µl ultra pure water and 5µl extracted DNA. Cycling conditions were as follows: denaturation at 98°C for 2min, 10 cycles of 95°C for 20s, 65°C for 15s, 70°C for 45s, followed by a final extension at 72°C for 5min and a 4°C hold. To reduce PCR- induced variation, this first round PCR was done in duplicates after which the products pooled (by mixing 10µl +10µl of each product on a clean plate) and purified on AMPure magnetic bead purification plate (according to manufacturer’s instructions). After purification, the second round PCR was prepared by mixing 9 µl of purified product with a 11 µl of master mix containing 10 µl KAPA Mastermix and 0.5+0.5 µl of forward and reverse Illumina Nextera barcoded indexing primers, added in unique combinations per each sample. The second PCR included 15 cycles with the same thermocycle conditions and was followed by the same AMPure purification step as the first PCR. Finally, amplified, indexed and purified 16S libraries were quantified for concentration (with Qubit), diluted to even concentration of 3-5 ng/ul and pooled for sequencing. Of each library preparation plate, the negative PCR control and 7 real samples (in diagonal order) were run on agrose gel in the end to ensure amplification success and lack of PCR-contamination. Extraction controls were not re-run on gels after amplification. Pooled libraries (x2) were sent off to the Centre for Genomic Research in Liverpool, where each pool was size-selected using a Pippin Prep, by excluding fragments outside the expected range. Libraries were then sequenced on two separate sequencing runs using 2x250bp paired-end sequencing on an Illumina MiSeq.

Bioinformatics and preprocessing of microbiota sequence data

Raw sequence data from each of the two sequencing batches was separately demultiplexed into their original samples and then processed through the DADA2 pipeline (version 1.14)(Callahan et al., 2016). Here, *cutadapt* (Martin, 2011) was first used to determine optimal trimming length to remove primer and adapter sequences. Based on this, 24 base pairs from the beginning of each read were trimmed away using the trimLeft argument in FilterAndTrim function in DADA2. As part of this function, and following visual inspection of sequence quality, low- quality tails were also trimmed, leaving 290 bp for forward and 230 bp for reverse reads. Next, sequences were dereplicated (identical sequences combined and their quality scores merged), amplicon sequence variants (ASVs) were

inferred using the DADA2 algorithm and reverse paired-end reads were merged. Reads that could not be merged or were of abnormal length (<365 bp or > 371 bp) were removed from the dataset.

After this step, data from the two sequencing batches were combined and processed together, using the R package *phyloseq* (McMurdie & Holmes, 2013). First, chimeric sequences were removed from the combined data and taxonomy was assigned to ASVs against the Silva Database (Quast et al., 2013; version 138). Following this, we used iNext package in R (Hsieh et al., 2016) to inspect rarefaction and sample completeness curves to determine a minimum threshold for sample read depth. We also found evidence of contamination in some of our extraction plates: There were bacterial DNA in our control samples and in some plates, there was a spatial autocorrelation signal in microbiota similarity among samples extracted in the same plate, i.e. samples extracted closer to each other were more likely to share contaminant ASVs. Contamination was much stronger in extraction controls, even ones that went through a clean (uncontaminated) PCR, implying that our DNA extraction protocol was vulnerable to contamination. This is a common issue with plate-format extraction kits, such as the ZYMO 96-well plate format faecal/soil DNA extraction kit we used (Minich et al., 2019). This kind of contamination likely happens through spillage between samples during the lysis step, and is prone to increase "technical noise" in the microbiota data. While this noise is random across biological groups in our data (as our samples were randomised across extraction plates, it can potentially diminish or mask some real biological differences between samples. We decided to mitigate this by

1) Subsetting data to contain only wood mouse faecal or soil samples and the remaining pre-processing steps were completed separately for these sample types.
2) Identifying potential contaminant ASVs with *decontam* algorithm (Davis et al., 2018) and filtering these out of the datasets.
3) Adding extraction plate and within-plate extraction distance as covariates in the models of microbiota similarity.

After this contamination mitigation step, we proceeded with processing the data by:

4) Removing singletons and doubletons, i.e. very rare taxa only present in abundance of <3 reads within sample,
5) Based on iNEXT completeness and rarefaction curves samples below read depth threshold (6000 reads for fecal samples, 800 for soil samples) were dropped out of the data as incomplete and unrepresentative.
6) Filtering away all ASVs assigned to Phylum "Cyanobacteria" or Family "Mitochondria", because these ASVs are likely not gut microbes but rather remnants of host DNA or plant chloroplasts from diet.

7) normalizing the read count data per sample as proportions.

The original number of unique sequence variants (ASVs) inferred by DADA2 algorithm was 6174 for wood mouse samples. After decontaminating the data, removing singletons and doubletons and all ASVs assigned to Cyanobacteria or Mitochondria, wood mouse samples had 1289 ASVs left.

Visualization of mock community composition revealed that our laboratory- and bioinformatics pipeline successfully captured the microbial diversity present in mock samples, though relative abundances varied somewhat from their theoretically true levels (Appendix 2 – Figure A below).

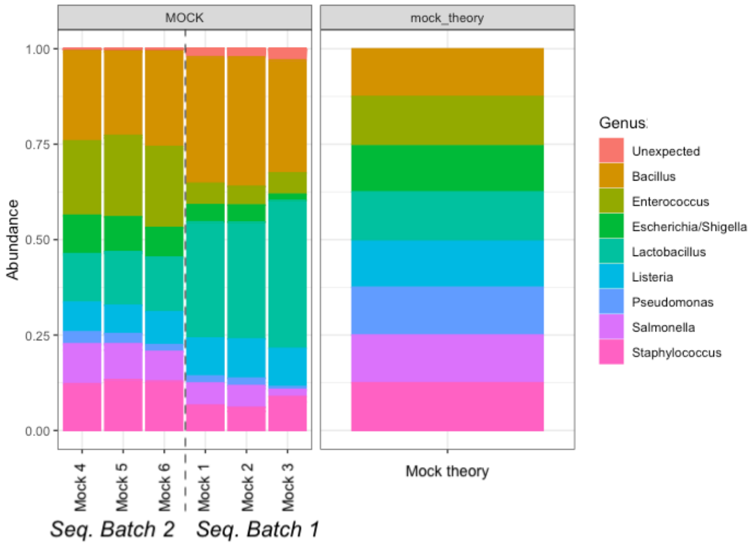

Appendix 2 – Figure A. Microbial standard (mock) community profiles.
Community composition in sequenced mock community samples compared to the expected composition (Mock theory).

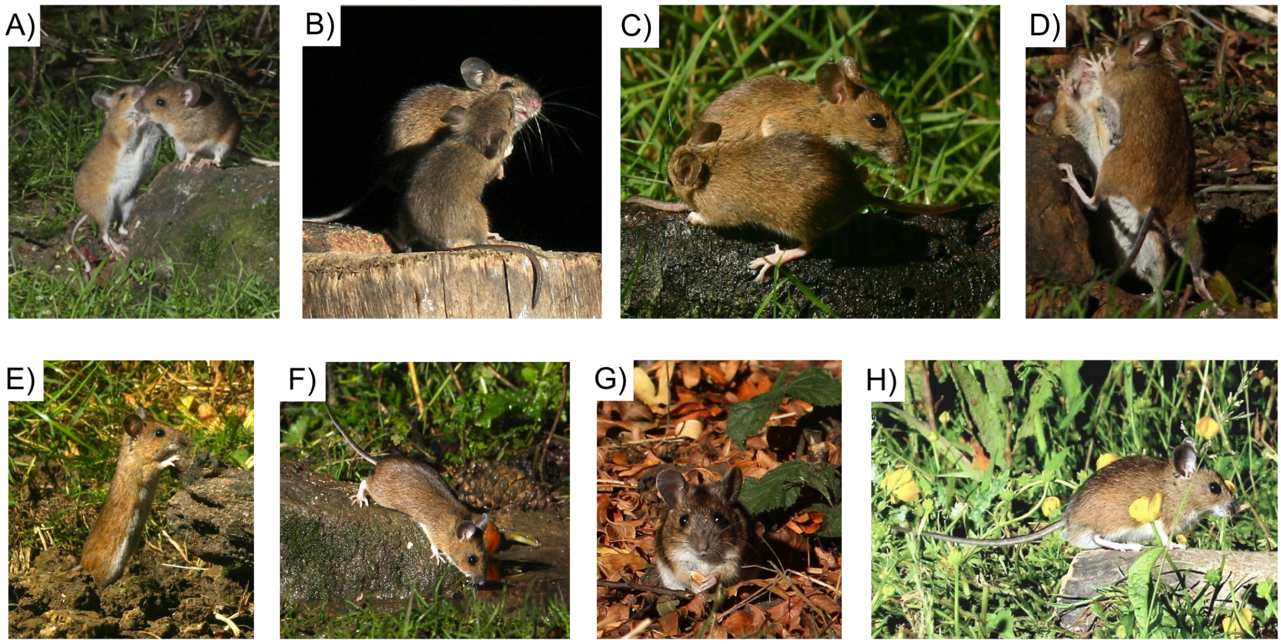

Supplementary Figure 1. Examples of social and environmental contact behaviours in wood mice. Social contact behaviours that could transmit gut microbes include licking and grooming (A-B), anogenital inspections (C) and aggressive interactions (D). Individual variation in environmental contacts that could transmit gut microbes include ranging behaviour and exposure to different microhabitats and soils (E-H). These photos were taken with a motion sensor camera trap (with a light) during natural wood mouse activity hours (night) from a natural wood mouse habitat in Milton Keynes, UK. Photo credit Roy and Marie Battell.

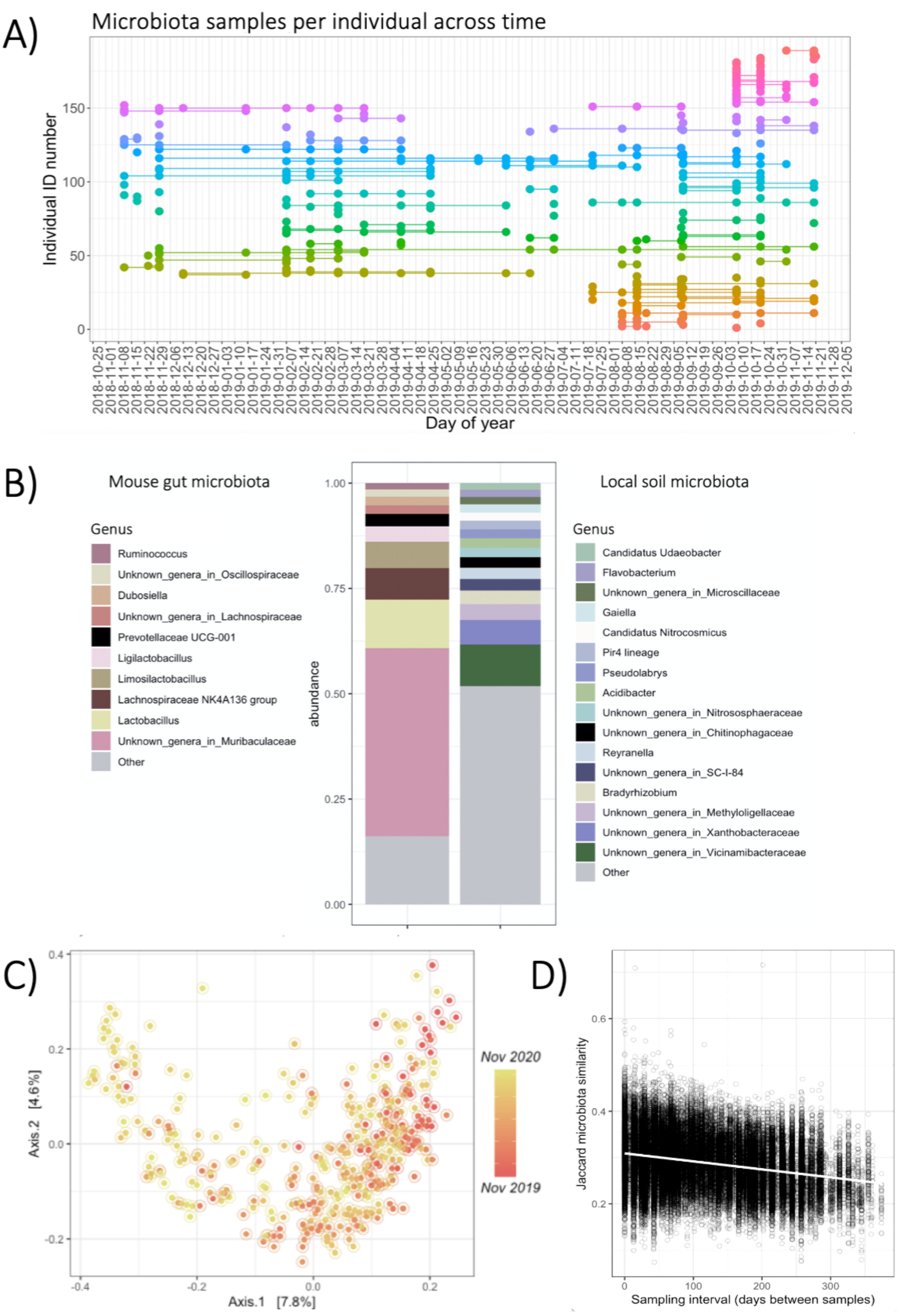

Supplementary Figure 2. Composition and variation in wood mouse gut microbiota and the local soil microbiota. A) Distribution of microbiota samples across time per individual mouse. B) Taxonomic composition of the most abundant (>1%) genera in the wood mouse gut microbiota (left) and local soil microbiota (right). C) PCoA plot illustrating how the primary axis of variation in wood mouse gut microbiota composition (Jaccard Index) reflects temporal changes in microbiota composition across the study period. D) Decay of microbiota similarity across time. Points are raw datapoints, line is a simple linear regression.

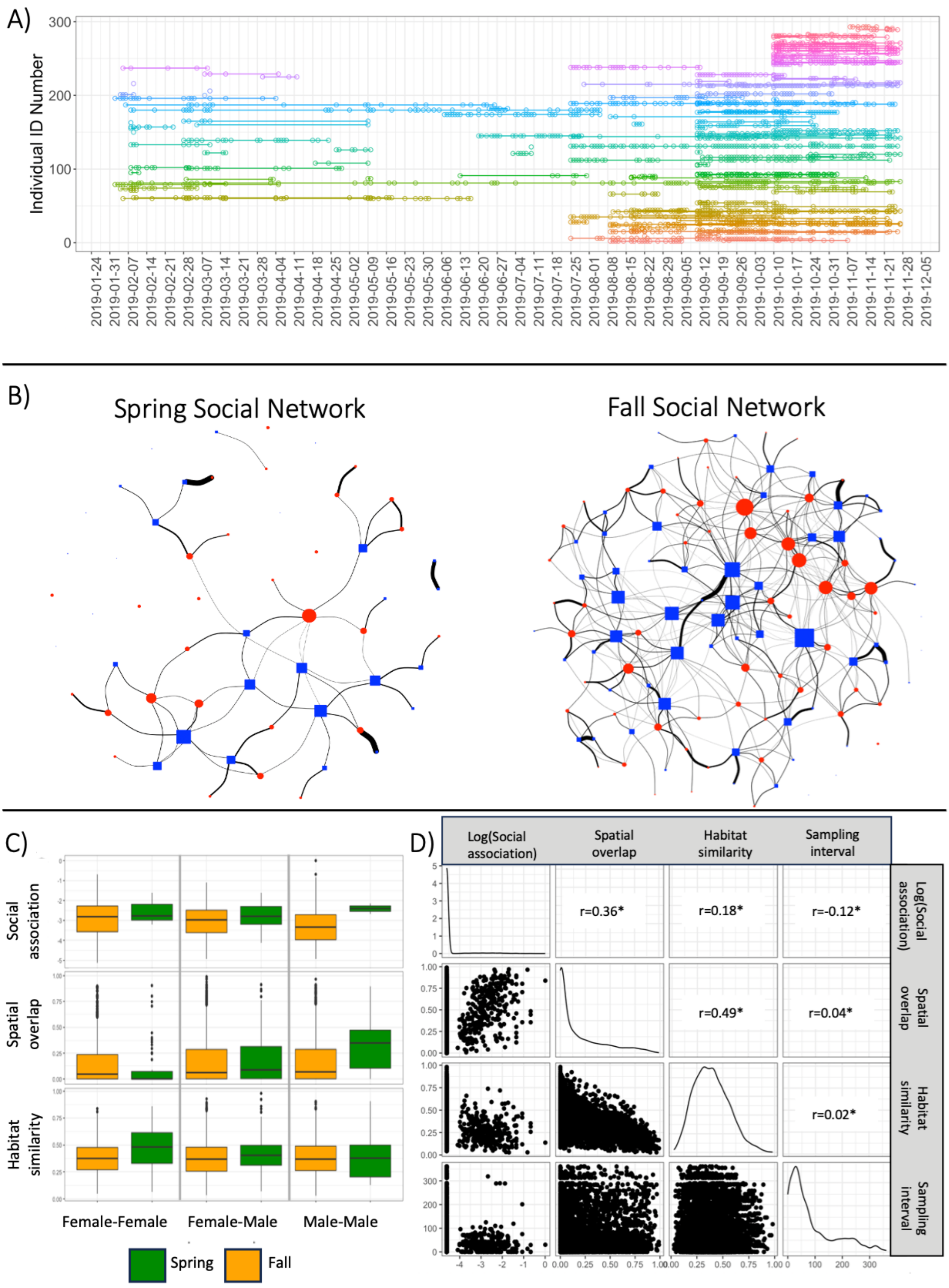
A)
Individual ID Number
B)
Spring Social Network
Fall Social Network
C)
Social association
Spatial overlap
Habitat similarity
Female-Female
Female-Male
Male-Male
Spring
Fall
D)
Log(Social association)
Spatial overlap
Habitat similarity
Sampling interval
r=0.36*
r=0.18*
r=-0.12*
r=0.49*
r=0.04*
r=0.02*

Supplementary Figure 3. Variation and covariation among social association, spatial overlap and habitat similarity. A) Active logging nights (nights when an individual was detected by a logger; dots connected by lines) per individual (colour, y-axis) across days of the year (x-axis). B) Social networks during spring and fall. C) Distribution of social association (upper panel, on a logarithmic scale), spatial overlap (middle panel) and habitat similarity (bottom panel) values across categories of sex-combinations (x-axis) and seasons (color), across 5356 pairwise comparisons among the 104 individual mice. Heavily left-skewed values of social association are reported on log-scale for ease of comparison. As per defaults in geom_boxplot - function in ggplot2 R package, horizontal lines denote medians, lower and upper hinges correspond to the first and third quartiles and whiskers extend from the hinge to the largest/smallest value no further away from the hinge than 1.5 times the distance between quartiles. Beyond this, outlier data points are plotted individually. D) Correlation plot (Pearson product-moment correlation coefficients) depicting associations among measures of social association (Adjusted SRI), spatial overlap (Bhattacaryya overlap between 75% kernel utilization distributions), habitat similarity (Bray-Curtis habitat similarity) and temporal distance (sampling interval). Lower triangle illustrates covariation among the two intersecting variables, diagonal depicts the distribution of raw values in the intersecting variable and upper triangle reports the correlation among the intersecting variables, as measured by Mantel test statistic.

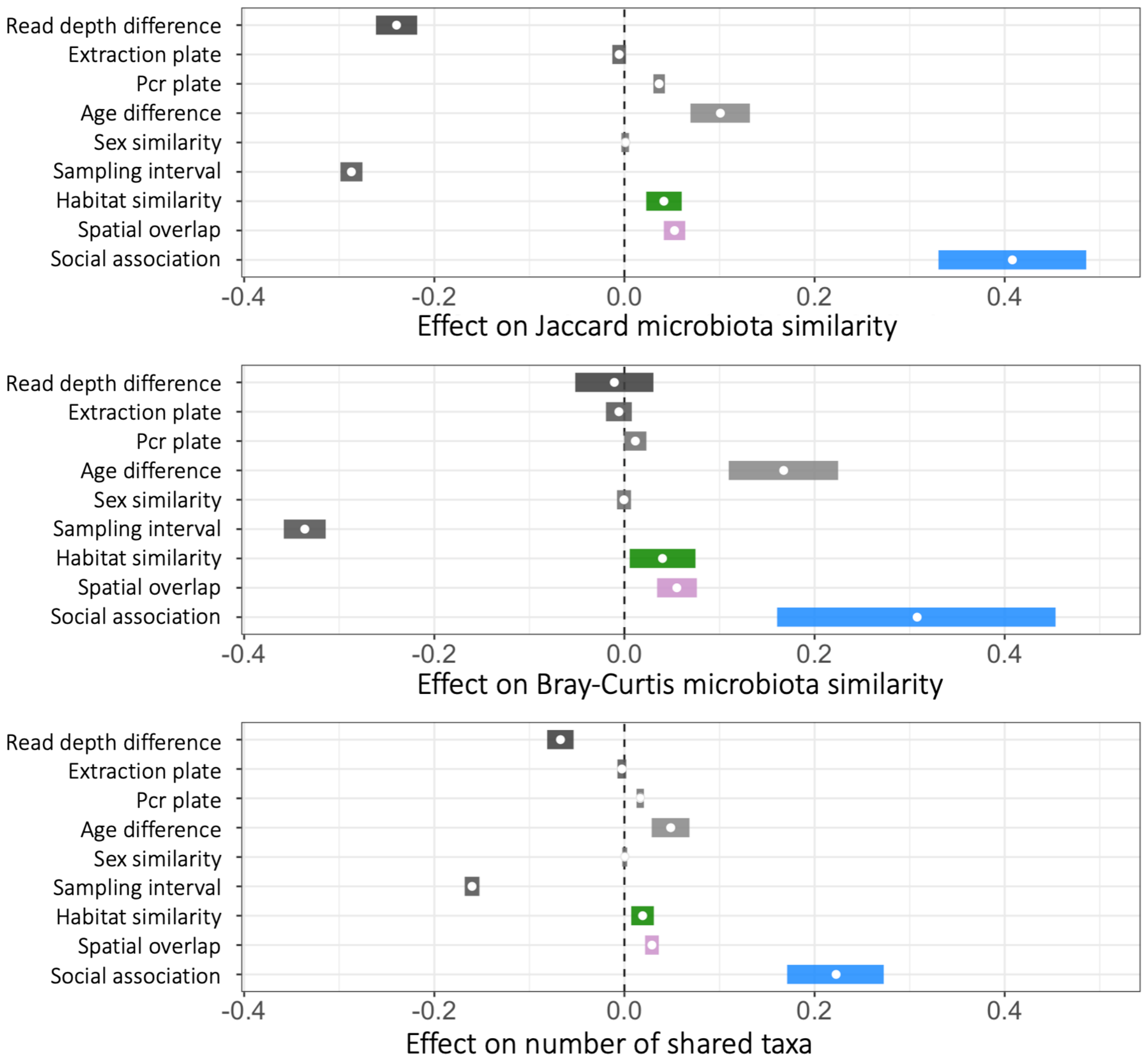

Supplementary Figure 4. Effects on alternative measures of microbiota similarity.

Results from dyadic Bayesian regression models (brms) predicting Jaccard microbiota similarity (top panel), Abundance-weighted Bray-Curtis microbiota similarity (middle panel) and number of shared taxa (bottom panel). Posterior means (points) and their 95% credible intervals (coloured lines) are plotted from models (Supplementary Tables 2B-C) with three alternative measures of pairwise microbiota similarity as the response. Where credible intervals do not overlap zero, a variable significantly predicts microbiota similarity while controlling for all other terms shown.

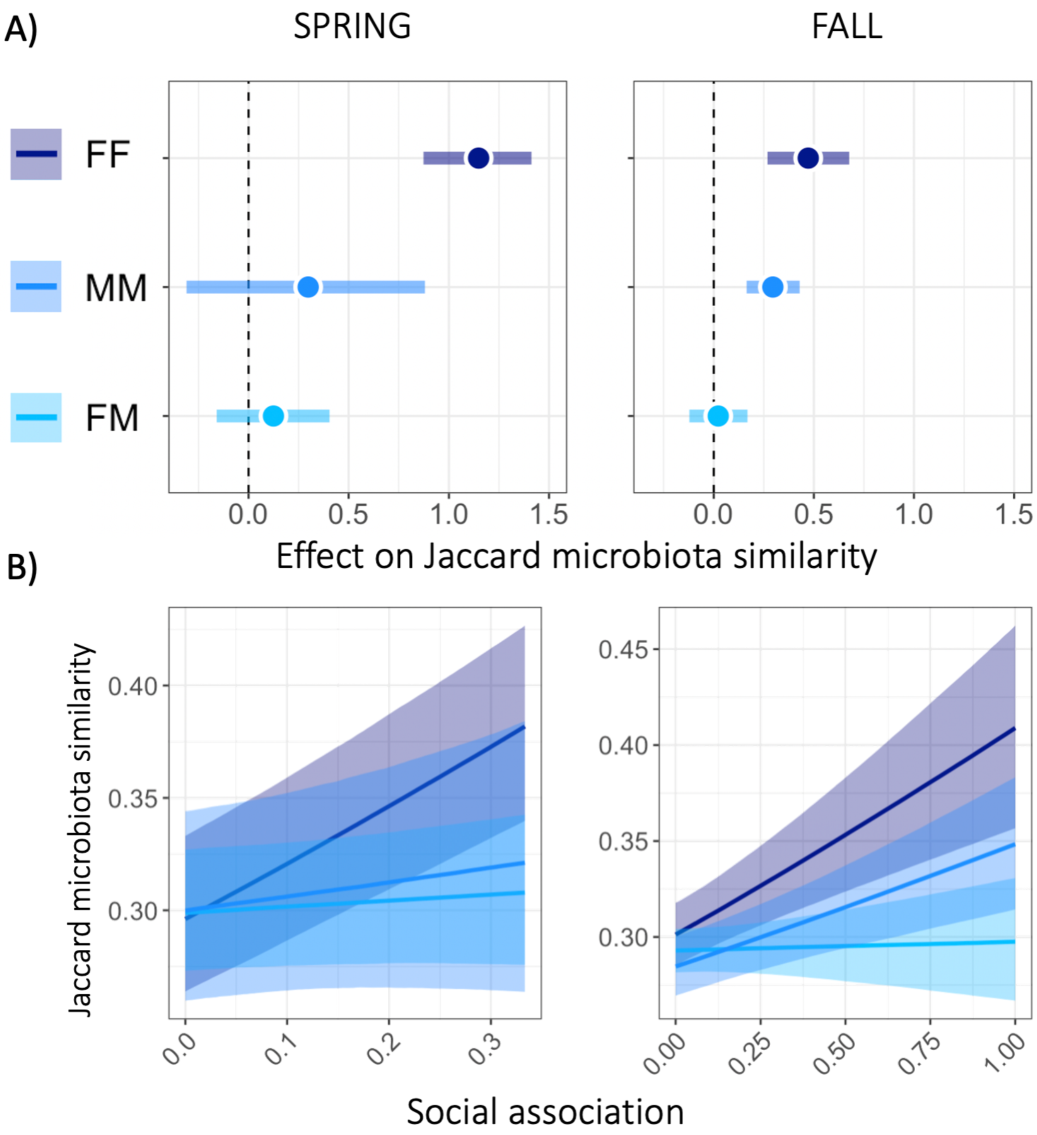

Supplementary Figure 5. Effects of social association on microbiota similarity across seasons and sexes. Social effect on Jaccard microbiota similarity (x-axis) in pairs with different sex-combinations (colours: FF=female-female, MM=male-male, FM=female-male), in spring (left panel) vs. fall (right panel). Posterior means (points) and their 95% credible intervals (coloured lines) are plotted from Bayesian regression (brms) models (Supplementary Tables 4A-B). Where credible intervals do not overlap zero, a social association significantly predicts microbiota similarity. B) Slopes of social association effect on microbiota similarity across sex combinations (colours) and seasons (panels), surrounded by 95% credible intervals (band).

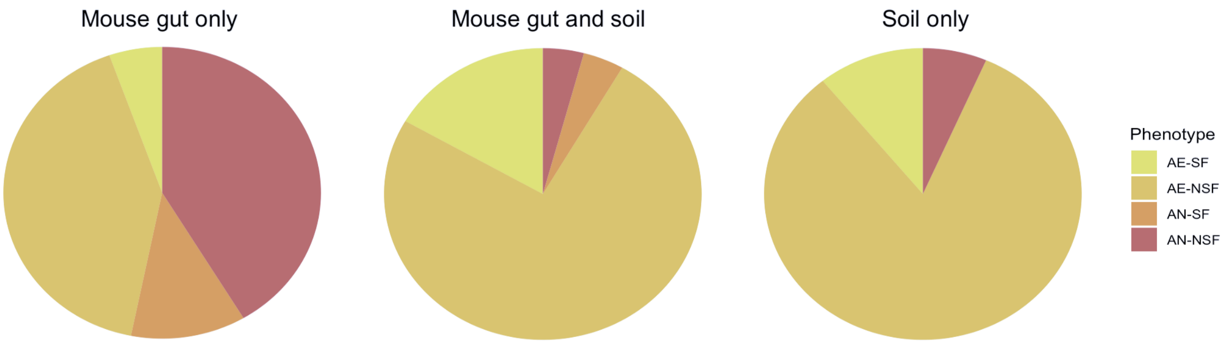

Supplementary Figure 6. Phenotype distribution among bacterial genera found in mouse gut, soil or both. Proportions of Aerobe spore-formers (AE-SF, yellow), aerobe non-spore-formers (AE-NSF, gold), anaerobe spore-formers (AN-SF, orange) and anaerobe non-spore-formers (AN-NSF, red) among genera with available phenotype info found i) only in mouse gut (n=111, left chart), ii) both in mouse gut and soil (n=24, middle chart), and iii) only in prevalent soil-only microbiota (n=46, right panel). Prevalent soil-only microbiota here comprises of a subset of all soil-only taxa, specifically the genera found from at least 50% of soil samples and is meant to serve as a representation of taxa common in the soil.

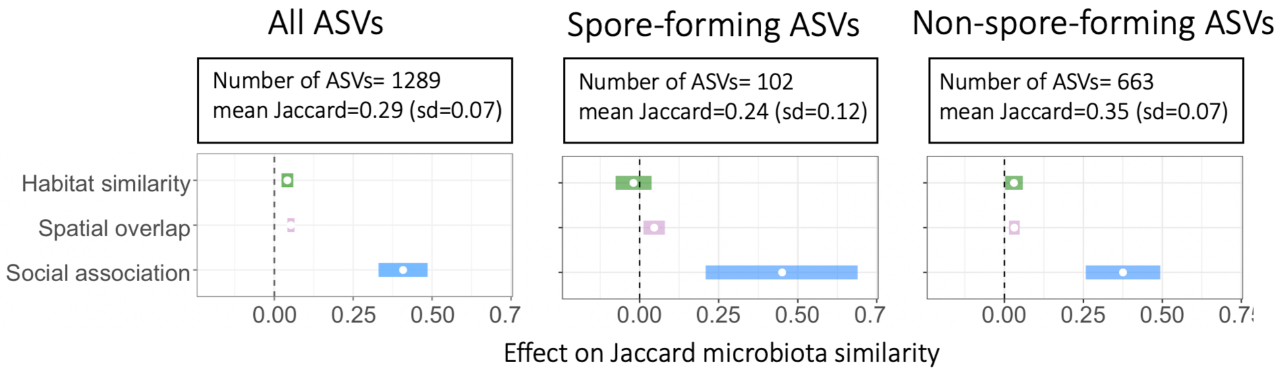

Supplementary Figure 7. Effects of main predictors on microbiota similarity in different phenotypic subsets of the microbiota – spore-formation. Effects of social association (blue), spatial overlap (purple) and habitat similarity (green) on microbiota similarity among all ASVs (left), spore-forming ASVs (middle) and non-spore-forming ASVs (right). Posterior means (points) and their 95% credible intervals (coloured lines) are plotted from Bayesian regression (brms) models (Supplementary Tables 5C-D) with pairwise microbiota similarity among hosts (Jaccard Index) as the response. Where credible intervals do not overlap zero, a variable significantly predicts microbiota similarity while controlling for all other terms shown.

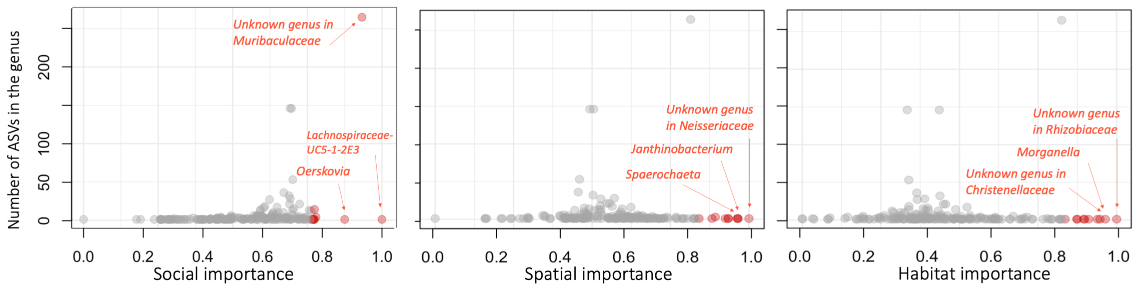

Genus	Phylum	no. ASVs	Importance
Lachnospiraceae-UC5-1-2E3	Firmicutes	1	1.00
Unknown genus in Muribaculaceae	Bacteroidota	265	0.93
Oerskovia	Actinobacteriota	1	0.87
Lachnospiraceae-A2	Firmicutes	4	0.78
Ruminococcus	Firmicutes	14	0.77
Blastocatella	Acidobacteriota	1	0.77
Candidatus Soleaferrea	Firmicutes	1	0.77
Allobaculum	Firmicutes	1	0.77
Gordonibacter	Actinobacteriota	1	0.77
Colidextribacter	Firmicutes	12	0.76
Mannheimia	Proteobacteria	1	0.76
Mesorhizobium	Proteobacteria	1	0.76
Lactobacillus	Firmicutes	13	0.76
Novosphingobium	Proteobacteria	1	0.75
Unknown genus in Thermomicrobiales	Chloroflexi	1	0.74
Anaerostipes	Firmicutes	4	0.74
Prevotellaceae Ga6A1 group	Bacteroidota	3	0.74
Vitreoscilla	Proteobacteria	1	0.74
Curtobacterium	Actinobacteriota	1	0.74
Eubacterium brachy group	Firmicutes	3	0.73

Genus	Phylum	no. ASVs	Importance
Unknown genus in Neisseriaceae	Proteobacteria	1	1.00
Janthinobacterium	Proteobacteria	1	0.97
Sphaerochaeta	Spirochaetota	1	0.97
Microvirga	Proteobacteria	1	0.96
Curtobacterium	Actinobacteriota	1	0.93
Vitreoscilla	Proteobacteria	1	0.93
Elusimicrobium	Elusimicrobiota	1	0.92
Rikenellaceae RC9 gut group	Bacteroidota	3	0.89
Unknown genus in Rhizobiaceae	Proteobacteria	1	0.88
Unknown genus in Coriobacteriales	Actinobacteriota	1	0.84
Variovorax	Proteobacteria	1	0.83
Pantoea	Proteobacteria	1	0.83
Peptococcus	Firmicutes	1	0.83
Unknown genus in Erysipelatoclostridiaceae	Firmicutes	1	0.82
Unknown genus in Muribaculaceae	Bacteroidota	265	0.81
Oerskovia	Actinobacteriota	1	0.81
Moraxella	Proteobacteria	1	0.79
Ferruginibacter	Bacteroidota	1	0.79
Puia	Bacteroidota	1	0.79
Polaromonas	Proteobacteria	1	0.77

Genus	Phylum	no. ASVs	Importance
Unknown genus in Rhizobiaceae	Proteobacteria	1	1.00
Morganella	Proteobacteria	1	0.96
Unknown genus in Christensenellaceae	Firmicutes	1	0.95
Candidatus Saccharimonas	Patescibacteria	1	0.94
Pantoea	Proteobacteria	1	0.91
Bilophila	Desulfobacterota	1	0.90
Allobaculum	Firmicutes	1	0.90
Polaromonas	Proteobacteria	1	0.87
Mannheimia	Proteobacteria	1	0.87
Acidibacter	Proteobacteria	1	0.84
Unknown genus in Muribaculaceae	Bacteroidota	265	0.82
Nakamurella	Actinobacteriota	1	0.82
Nocardioides	Actinobacteriota	1	0.82
Oscillospiraceae-UCG-005	Firmicutes	1	0.82
Massilia	Proteobacteria	1	0.82
Paraprevotella	Bacteroidota	1	0.82
Amaricoccus	Proteobacteria	1	0.79
Ochroba trum	Proteobacteria	1	0.79
Candidatus Soleaferrea	Firmicutes	1	0.79
Actinoplanes	Actinobacteriota	2	0.78

Supplementary Figure 8. Overview of the most important genera for social, spatial and habitat signal in microbiota similarity. Top panel: Distribution of social (left), spatial (middle) and habitat (right) importance scores (x-axis) against the number of ASVs in each genus (y-axis). Top 10 most important genera are marked red. Bottom table: Overview of top 20 most important genera in each importance type. Here too, top 10 most important genera in each category are marked red.

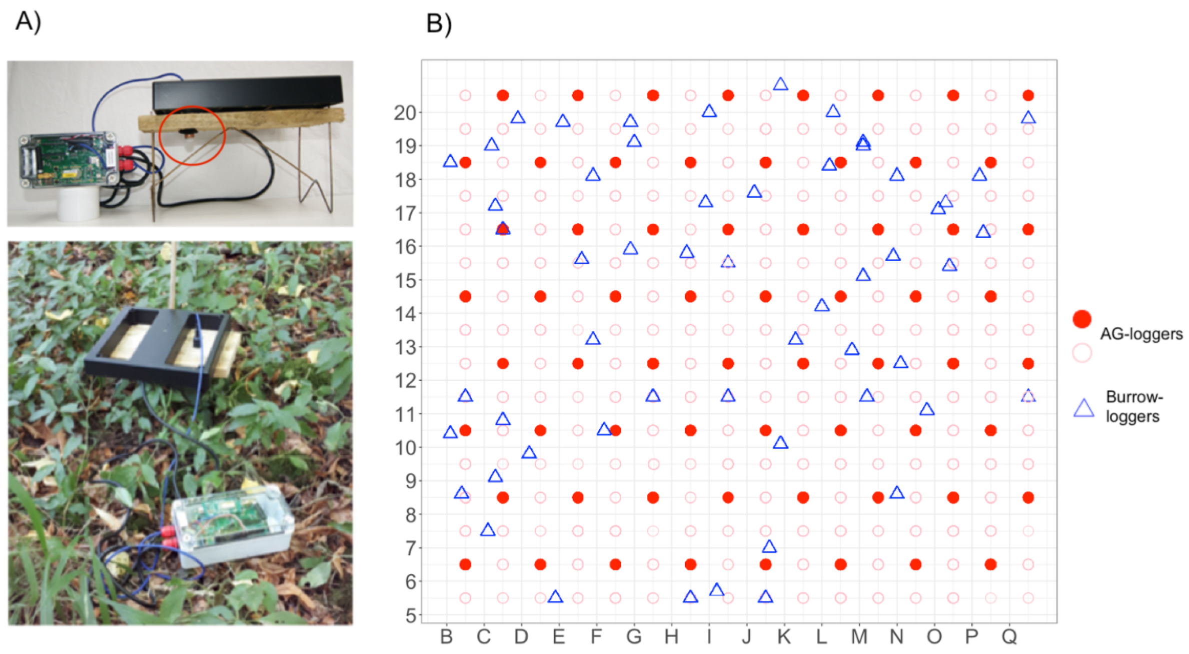

Supplementary Figure 9. The RFID logger device and tracking protocol

A) The logger was un-baited and consisted of a motion sensor (red circle, top figure), that woke up the detection coil (black square) whenever a warm object, such as a rodent came nearby. The coil, sitting on a wooden “table” to allow rodents to pass under as well as around it, then created a ~1 m2 oscillating detection field that would interfere – and subsequently log- any 124 kHz RFID tag within this field. Exploiting this, wild rodents were tagged with a subcutaneous passive integrated transponder (PIT-) tags containing a 10-digit HEX-code identification number readable in this exact oscillation frequency. When a rodent tag was read by the coil, this identification number was saved on an SD-card (in the grey box) and additionally transmitted into and interactive internet database through an antenna (in the grey box) connected to the broadband network covering Wytham Woods (Wytham Data Net). B) To collect data for social networks, 60 “above-ground” loggers (red dots) were positioned in an even chequerboard design, where loggers would be in the middle of every fourth 10 x 10 m grid cell across the 2.56 study grid. Additionally, loggers were fortnightly rotated throughout the whole study (February-November 2019) by moving each logger one cell (=10 m) north (increasing numbers in y axis). This way each grid cell had a logger (pink circles) for two weeks every two months.

In addition to these 60 loggers, for home range analysis, we additionally used data from an extra set of 60 Burrow-loggers (blue triangles), which were positioned approximately evenly across the grid, on top of a set of known active mouse burrows across the grid from July until November, and not rotated. Burrow-logger data was used to complement AG-logger data in home-range estimation, but not in any other logger-derived measures.

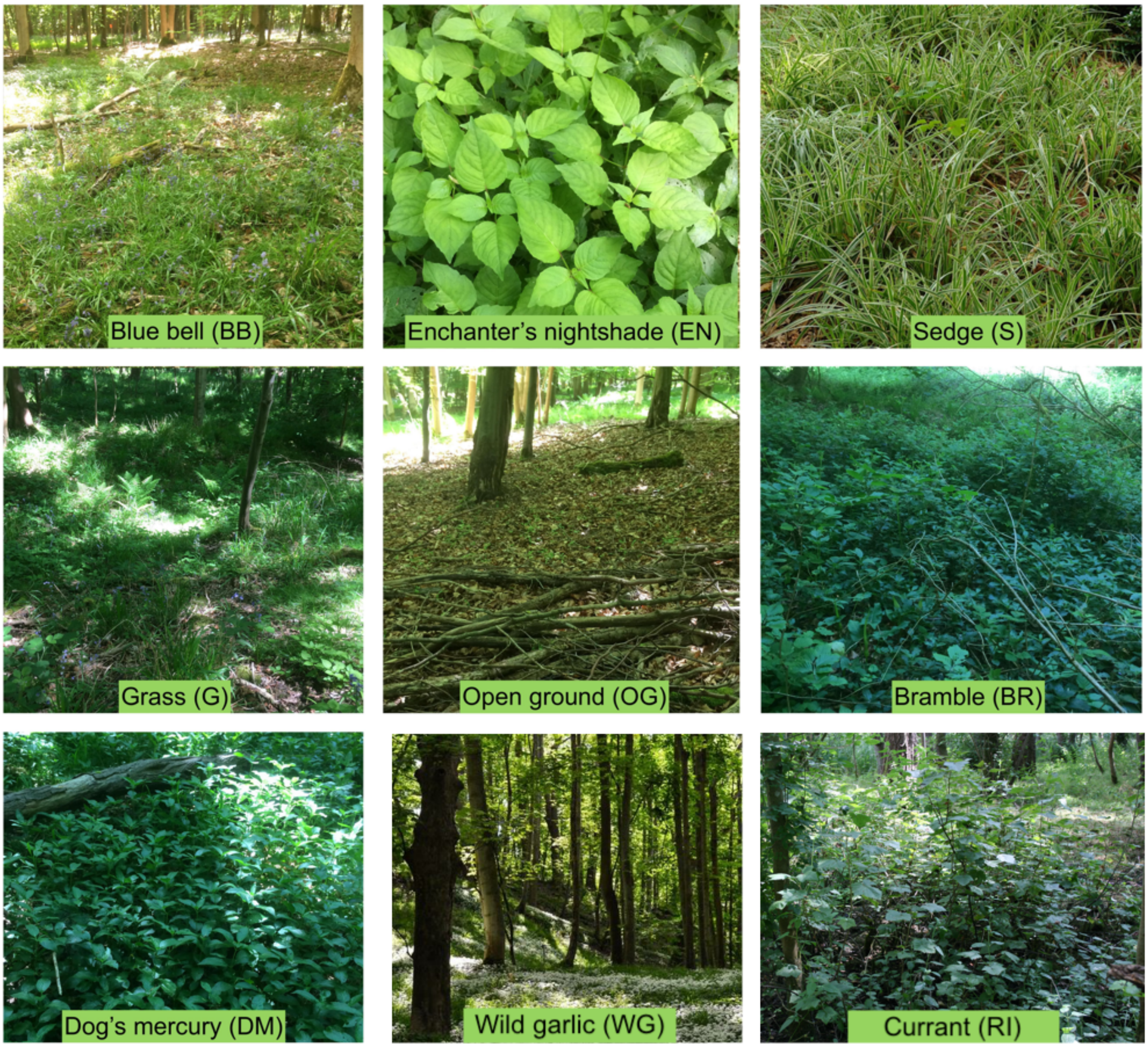

Supplementary Figure 10: **Main ground cover habitat types in Holly Hill study site.**

Photos are taken from Wytham Woods. All photos by authors, except “Currant”-photo, which is by Keith Kirby.

Supplementary Table 1. Model results: Effects of Social association, spatial overlap and habitat similarity on microbiota similarity.

Results of *brms* models testing the effect of social association, spatial overlap, habitat similarity and covariates on different measures of microbiota similarity: A) Jaccard Index (betaregression), B) Bray-Curtis similarity (betaregression), C) number of shared taxa (poisson regression). Significant terms (where 95% credible intervals do not include zero) are shown in bold. Est.Error indicates the standard deviation of the posterior distribution.

**Supplementary Table 1A. Predicting Jaccard microbiota similarity**				
	Estimate	Est.Error	l-95% CI	u-95% CI
Intercept	-0.93	0.03	-0.99	-0.88
**Sample read depth difference**	-0.24	0.01	-0.26	-0.22
Extraction distance	-0.01	0.00	-0.01	0.00
**PCR-plate similarity**	0.04	0.00	0.03	0.04
**Age-similarity**	0.10	0.02	0.07	0.13
Sex similarity	0.00	0.00	-0.00	0.00
**Sampling interval**	-0.29	0.01	-0.30	-0.28
**Habitat similarity**	0.04	0.01	0.02	0.06
**Spatial overlap**	0.05	0.01	0.04	0.06
**Social association**	0.41	0.04	0.33	0.49
**Supplementary Table 1B. Predicting Bray-Curtis microbiota similarity**				
	Estimate	Est.Error	l-95% CI	u-95% CI
Intercept	-1.09	0.05	-1.19	-1.00
**Sample read depth difference**	-0.01	0.02	-0.05	0.03
Extraction distance	-0.01	0.01	-0.02	0.01
**PCR-plate similarity**	0.01	0.01	-0.00	0.02
**Age-similarity**	0.17	0.03	0.11	0.22
Sex similarity	-0.00	0.00	-0.01	0.01
**Sampling interval**	-0.34	0.01	-0.36	-0.31
**Habitat similarity**	0.04	0.02	0.01	0.07
**Spatial overlap**	0.05	0.01	0.03	0.08
**Social association**	0.31	0.08	0.16	0.45
**Supplementary Table 1C. Predicting number of shared taxa**				
	Estimate	Est.Error	l-95% CI	u-95% CI
Intercept	4.36	0.03	4.30	4.42
**Sample read depth difference**	-0.07	0.01	-0.08	-0.05
Extraction distance	-0.00	0.00	-0.01	0.00
**PCR-plate similarity**	0.02	0.00	0.01	0.02

**Age-similarity**	0.05	0.01	0.03	0.07
Sex similarity	0.00	0.00	-0.00	0.00
**Sampling interval**	-0.16	0.00	-0.17	-0.15
**Habitat similarity**	0.03	0.01	0.01	0.03
**Spatial overlap**	0.02	0.00	0.02	0.04
**Social association**	0.22	0.03	0.17	0.27

Supplementary Table 2. Model results: Effects of social association, spatial overlap and habitat similarity on microbiota similarity among pairs with different sex categories.

Results of *brms* models testing whether effects of social association, spatial overlap and habitat similarity on microbiota similarity (Jaccard Index) varied according to the sex combination of mouse pairs, while accounting for covariates. Significant terms are those where 95% credible intervals do not include zero. Est.Error indicates the standard deviation of the posterior distribution. Interaction effects are reported as slopes relative to the base category of female-female as per model output.

**Supplementary Table 2. Effects on microbiota similarity across sex combinations**				
	Estimate	Est.Error	l-95% CI	u-95% CI
Intercept	-0.90	0.04	-0.97	-0.82
Sample read depth difference	-0.24	0.01	-0.26	-0.22
Extraction distance	-0.01	0.00	-0.01	0.00
PCR-plate similarity	0.04	0.00	0.03	0.04
Age similarity	0.10	0.02	0.07	0.13
Sampling interval	-0.29	0.01	-0.30	-0.28
Habitat similarity	0.02	0.02	-0.01	0.05
Sex category (female-male)	-0.03	0.03	-0.08	0.03
Sex category (male-male)	-0.10	0.05	-0.20	0.00
Spatial overlap	0.10	0.01	0.07	0.12
Social association	0.74	0.08	0.59	0.90
Habitat similarity: Sex category (female-male)	0.00	0.02	-0.03	0.04
Habitat similarity: Sex category (male-male)	0.07	0.02	0.02	0.11
Spatial overlap: Sex category (female-male)	-0.05	0.01	-0.08	-0.02
Spatial overlap: Sex category (male-male)	-0.07	0.02	-0.10	-0.04
Social association: Sex category (female-male)	-0.56	0.10	-0.76	-0.37
Social association: Sex category (male-male)	-0.34	0.10	-0.54	-0.14

Supplementary Table 3. Model results: Effects of spring and fall social association on microbiota similarity among pairs with different sex categories.

Results of *brms* models testing the effects of spring and fall social association across sex combinations on microbiota similarity (Jaccard Index), while accounting for covariates. Age is not included as a covariate in the spring model, as there was not enough age variation in the spring (only adults). Significant terms (where 95% credible intervals do not include zero) are shown in bold. Est.Error indicates the standard deviation of the posterior distribution. For ease of interpretation, interaction effects between sex combination and social association are *NOT* reported as slopes relative to each other but as independent effects within each group (e.g., "Male-male Spring social association" means the independent effect of social association on microbiota among males).

**Supplementary Table 3A. Spring**				
	Estimate	Est.Error	l-95% CI	u-95% CI
Intercept	-0.81	0.09	-0.97	-0.64
**Sample read depth difference**	-0.21	0.04	-0.30	-0.13
Extraction distance	0.00	0.01	-0.02	0.02
**PCR-plate similarity**	0.02	0.01	0.00	0.04
**Sampling interval**	-0.52	0.03	-0.57	-0.46
**Habitat similarity**	0.14	0.03	0.07	0.21
Spatial overlap	-0.02	0.02	-0.07	0.02
Sex category (female-male)	0.01	0.07	-0.12	0.14
Sex category (male-male)	0.02	0.13	-0.25	0.28
**Female-female Spring social association**	1.15	0.14	0.88	1.42
Female-male Spring social association	0.12	0.19	-0.16	0.40
Male-male Spring social association	0.30	0.32	-0.31	0.88
**Supplementary Table 3B. Fall**				
	Estimate	Est.Error	l-95% CI	u-95% CI
Intercept	-0.91	0.04	-1.00	-0.83
**Sample read depth difference**	-0.24	0.01	-0.27	-0.21
Extraction distance	-0.01	0.00	-0.01	0.00
**PCR-plate similarity**	0.03	0.00	0.03	0.04
**Age similarity**	0.11	0.02	0.08	0.14
**Sampling interval**	-0.21	0.02	-0.24	-0.19
**Habitat similarity**	0.03	0.01	0.01	0.06
**Spatial overlap**	0.10	0.01	0.08	0.11
Sex category (female-male)	-0.04	0.03	-0.09	0.01
Sex category (male-male)	-0.08	0.05	-0.19	0.03
**Female-female Fall social association**	0.47	0.10	0.27	0.68
Female-male Fall social association	0.02	0.12	-0.12	0.17
**Male-male Fall social association**	0.30	0.12	0.16	0.43

Supplementary Table 4. Aerotolerance and spore-forming ability of bacterial genera

This table is attached as a separate csv file “Supplementary_Table_4.csv”. We used Bergey’s Manual of Systematics of Archaea and Bacteria alongside other individual scientific publications to find information about the aerotolerance and spore-formation abilities of bacterial genera present in our data. References mentioned in the table are included in the Supplementary References in the end of this document. Specifically, we searched information for the 188 genera present in the mouse gut or mouse gut and soil and additionally 111 genera present only in the soil. For these soil-only taxa, we only considered those which were present in at least half of the soil samples. For genera with mixed or unknown aerotolerance or sporulation, we labelled “unknown”. For unknown genera within a known family, we were conservative in that we generally considered them as having unknown aerotolerance and spore-formation. However, when other members of the family were consistently of a given phenotype, we gave a genus the phenotype of its family. For example, since all described strains in the family Muribaculaceae are anaerobic and non-spore-forming, we considered “unknown genus in Muribaculaceae” to also be anaerobic and non-spore-forming. For some genera with missing information on these phenotypic traits, we also labelled them after another name for the same genus present in the literature (e.g., “UBA1819” as “Faecalibacterium”) or according to the genus that they had been previously classified as belonging to (e.g., *Lysinibacillus* as part of *Bacillus*). All genera assigned an aerotolerance or spore-formation label in any of these indirect manners, have been marked in the columns “Aerotolerance_indirectly_inferred” and “Sporeformation_indirectly_inferred”, and the taxon after which they were labelled is specified in the “References” column. Spore-formation column labels taxa as spore-forming (SF) or non-spore-forming (NSF). Aerotolerance column labels taxa as anaerobic (=obligate anaerobes) or aerotolerant (all facultative anaerobes, microaerobes, or aerobes). As in the analyses, as in Suzuki et al., 2019, all taxa with any aerotolerance (all except obligate anaerobes) where considered aerotolerant.

Supplementary Table 5. Model results: Effects on microbiota similarity in different phenotypic subsets of microbiota.

Results of brms models testing the effect of social association, spatial overlap, habitat similarity and covariates on gut microbiota similarity (Jaccard Index) of a) aerotolerant taxa or b) anaerobic taxa, c) spore-forming taxa and d) non-spore-forming taxa. Significant terms (where 95% credible intervals do not include zero) are shown in bold. Est.Error indicates the standard deviation of the posterior distribution. Note that posterior distributions are not directly comparable between models due to inherently different uncertainty, intercepts and slopes across phenotypic data subsets.

**Supplementary Table 5 Aerotolerant microbiota**				
	Estimate	Est.Error	l-95% CI	u-95% CI
Intercept	-1.08	0.10	-1.27	-0.88
**Sample read depth difference**	-0.42	0.04	-0.50	-0.34
Extraction distance	-0.05	0.01	-0.08	-0.03
**PCR-plate similarity**	0.26	0.01	0.24	0.28
Age-similarity	0.10	0.06	-0.03	0.22
**Sex similarity**	-0.01	0.01	-0.02	0.01
**Sampling interval**	-0.76	0.02	-0.81	-0.72
Habitat similarity	-0.03	0.04	-0.10	0.04
**Spatial overlap**	0.07	0.02	0.03	0.11
Social association	0.17	0.15	-0.12	0.46
**Supplementary Table 5B. Anaerobic microbiota**				
	Estimate	Est.Error	l-95% CI	u-95% CI
Intercept	-1.17	0.04	-1.25	-1.09
**Sample read depth difference**	-0.29	0.02	-0.33	-0.25
Extraction distance	0.00	0.01	-0.01	0.02
**PCR-plate similarity**	0.02	0.01	0.01	0.03
**Age-similarity**	0.09	0.03	0.04	0.14
Sex similarity	0.00	0.00	0.00	0.01
**Sampling interval**	-0.29	0.01	-0.31	-0.27
**Habitat similarity**	0.04	0.02	0.00	0.07
**Spatial overlap**	0.03	0.01	0.01	0.05
**Social association**	0.42	0.07	0.28	0.56
**Supplementary Table 5C Spore-forming microbiota**				
	Estimate	Est.Error	l-95% CI	u-95% CI
Intercept	-1.24	0.08	-1.40	-1.08
**Sample read depth difference**	-0.05	0.03	-0.11	0.02
Extraction distance	0.00	0.01	-0.03	0.02
**PCR-plate similarity**	0.00	0.01	-0.01	0.02

Age-similarity	0.05	0.05	-0.05	0.14
**Sex similarity**	0.02	0.01	0.00	0.03
**Sampling interval**	-0.22	0.02	-0.25	-0.18
Habitat similarity	-0.02	0.03	-0.08	0.04
**Spatial overlap**	0.05	0.02	0.01	0.08
Social association	0.45	0.12	0.21	0.69
**Supplementary Table 5D. Non-spore-forming microbiota**				
	Estimate	Est.Error	l-95% CI	u-95% CI
Intercept	-1.01	0.04	-1.08	-0.94
**Sample read depth difference**	-0.34	0.02	-0.37	-0.30
Extraction distance	-0.01	0.01	-0.02	0.00
**PCR-plate similarity**	0.06	0.00	0.05	0.07
**Age-similarity**	0.10	0.02	0.06	0.15
Sex similarity	0.00	0.00	-0.01	0.01
**Sampling interval**	-0.38	0.01	-0.40	-0.36
**Habitat similarity**	0.03	0.01	0.00	0.06
**Spatial overlap**	0.03	0.01	0.01	0.05
**Social association**	0.38	0.06	0.26	0.49

Supplementary Table 6. Model results: Effects of Phenotypes on importance scores across bacterial genera.

Results of three brms models testing the effect of binary Aerotolerance and Sporulation (0/1) on importance scores of social (model 6A), spatial (model 6B) and habitat (model 6C) signal across bacterial genera, while controlling for their phylogeny. Significant terms (where 95% credible intervals do not overlap zero) are shown in bold. Est.Error indicates the standard deviation of the posterior distribution. Model formulas were:

Social Importance~ Aerotolerance*Sporulation + (1 | gr(genus, cov = Phylogenetic tree).

Spatial Importance~ Aerotolerance*Sporulation + (1 | gr(genus, cov = Phylogenetic tree).

Habitat Importance~ Aerotolerance*Sporulation + (1 | gr(genus, cov = Phylogenetic tree).

Here, interaction effects are reported as slopes relative to each other as per model output.

**Supplementary Table 6A. Social Importance**				
	Estimate	Est.Error	l-95% CI	u-95% CI
Intercept	0.58	0.06	0.43	0.67
**Aerotolerance**	**-0.07**	**0.04**	**-0.14**	**-0.00**
Sporulation	-0.05	0.06	-0.16	0.06
Aerotolerance:Sporulation	0.08	0.08	-0.08	0.24
**Supplementary Table 6B. Spatial Importance**				
	Estimate	Est.Error	l-95% CI	u-95% CI
Intercept	0.48	0.10	0.24	0.61
Aerotolerance	-0.02	0.04	-0.10	0.06
Sporulation	0.02	0.06	-0.10	0.13
Aerotolerance:Sporulation	0.13	0.08	-0.03	0.28
**Supplementary Table 6C. Habitat Importance**				
	Estimate	Est.Error	l-95% CI	u-95% CI
Intercept	0.47	0.06	0.33	0.58
Aerotolerance	-0.02	0.05	-0.12	0.07
**Sporulation**	**-0.17**	**0.07**	**-0.31**	**-0.02**
**Aerotolerance:Sporulation**	**0.21**	**0.10**	**0.00**	**0.41**

Supplementary Table 7. Model results: Effects of microbial combination phenotypes on importance scores across bacterial genera. Results of three brms models testing the effect of bacterial phenotype (4-level factor: aerotolerant spore-former AE-SF, aerotolerant non-spore-former AE-NSF, anaerobic spore-former AN-SF, anaerobic non-spore-former AN-NSF) on importance scores of social (model 7A), spatial (model 7B) and habitat (model 7C) signal across bacterial genera, while controlling for their phylogeny. Significant terms are those where 95% credible intervals do not include zero. Est.Error indicates the standard deviation of the posterior distribution. Model formulas were:

Social Importance ~ phenotype+ (1 | gr(genus, cov = Phylogenetic tree)

Spatial Importance ~ phenotype+ (1 | gr(genus, cov = Phylogenetic tree)

Habitat Importance ~ phenotype+ (1 | gr(genus, cov = Phylogenetic tree)

**Supplementary Table 7A: Social importance**				
	Estimate	Est.Error	l-95% CI	u-95% CI
Intercept	0.58	0.06	0.43	0.67
Phenotype (AN-SF)	-0.05	0.06	-0.16	0.07
Phenotype (AE-NSF)	-0.07	0.04	-0.15	-0.00
Phenotype (AE-SF)	-0.04	0.06	-0.16	0.07
**Supplementary Table 7B: Spatial importance**				
	Estimate	Est.Error	l-95% CI	u-95% CI
Intercept	0.61	0.10	0.37	0.76
Phenotype (AN-NSF)	-0.12	0.06	-0.24	-0.00
Phenotype (AN-SF)	-0.10	0.08	-0.25	0.04
Phenotype (AE-NSF)	-0.14	0.06	-0.25	-0.03
**Supplementary Table 7C: Habitat importance**				
	Estimate	Est.Error	l-95% CI	u-95% CI
Intercept	0.48	0.06	0.36	0.59
Phenotype (AN-SF)	-0.22	0.08	-0.38	-0.06
Phenotype (AE-NSF)	-0.02	0.05	-0.11	0.07
Phenotype (AE-SF)	0.05	0.08	-0.11	0.21

Supplementary Table 8. Model results: Results of post hoc models testing whether specific phenotype categories differ significantly from other genera in their importance. Results of three brms models, testing whether A) Anaerobe non-spore formers (AN-NSF) have significantly higher social importance than other genera, B) Aerobe-spore formers (AE-SF) have significantly higher spatial importance than other genera and C) Anaerobe spore formers (AN-SF) have significantly lower habitat importance than other genera. Predictor term was a binary variable describing whether a genus was of a given phenotype (1) or not (0). Significant terms (where 95% credible intervals do not include zero) are shown in bold. Est.Error indicates the standard deviation of the posterior distribution. Model formulas were:

Social Importance ~ AN-NSF + (1 | gr(genus, cov = Phylogenetic tree)

Spatial Importance ~ AE-SF + (1 | gr(genus, cov = Phylogenetic tree)

Habitat Importance ~ AN-SF + (1 | gr(genus, cov = Phylogenetic tree)

**Supplementary Table 8A: Social importance**				
	Estimate	Est.Error	l-95% CI	u-95% CI
Intercept	0.51	0.06	0.37	0.60
Phenotype (AN-NSF)	0.06	0.03	-0.00	0.13
**Supplementary Table 8B: Spatial importance**				
	Estimate	Est.Error	l-95% CI	u-95% CI
Intercept	0.47	0.09	0.25	0.59
**Phenotype (AE-SF)**	**0.13**	**0.05**	**0.03**	**0.24**
**Supplementary Table 8C: Habitat importance**				
	Estimate	Est.Error	l-95% CI	u-95% CI
Intercept	0.46	0.05	0.34	0.55
**Phenotype (AN-SF)**	-0.16	0.07	-0.30	-0.02

Supplementary References

Adeolu, M., Alnajar, S., Naushad, S., & Gupta, R. S. (2016). Genome-based phylogeny and taxonomy of the 'Enterobacteriales': proposal for Enterobacterales ord. nov. divided into the families Enterobacteriaceae, Erwiniaceae fam. nov., Pectobacteriaceae fam. nov., Yersiniaceae fam. nov., Hafniaceae fam. nov., Morganellaceae fam. nov., and Budviciaceae fam. nov. *International journal of systematic and evolutionary microbiology*, *66*(12), 5575-5599.

Amir, I., Bouvet, P., Legeay, C., Gophna, U., & Weinberger, A. (2014). Eisenbergiella tayi gen. nov., sp. nov., isolated from human blood. International journal of systematic and evolutionary microbiology, 64(Pt_3), 907-914.

Baron, S. (Ed.), Medical microbiology (4th edition), University of Texas Medical Branch, Galveston, TX (1996)

Benga, L., Sager, M., & Christensen, H. (2018). From the [Pasteurella] pneumotropica complex to Rodentibacter spp.: an update on [Pasteurella] pneumotropica. In *Veterinary Microbiology* (Vol. 217, pp. 121–134). Elsevier B.V. https://doi.org/10.1016/j.vetmic.2018.03.011

Brady, C., Cleenwerck, I., Venter, S., Coutinho, T., & de Vos, P. (2013). Taxonomic evaluation of the genus Enterobacter based on multilocus sequence analysis (MLSA): Proposal to reclassify E. nimipressuralis and E. amnigenus into Lelliottia gen. nov. as Lelliottia nimipressuralis comb. nov. and Lelliottia amnigena comb. nov., respectively, E. gergoviae and E. pyrinus into Pluralibacter gen. nov. as Pluralibacter gergoviae comb. nov. and Pluralibacter pyrinus comb. nov. *Systematic and Applied Microbiology*, *36*(5), 309–319. https://doi.org/10.1016/j.syapm.2013.03.005

Browne, H. P., Forster, S. C., Anonye, B. O., Kumar, N., Neville, B. A., Stares, M. D., Goulding, D., & Lawley, T. D. (2016). Culturing of "unculturable" human microbiota reveals novel taxa and extensive sporulation. *Nature*, *533*(7604), 543–546. https://doi.org/10.1038/nature17645

Cai, S., & Dong, X. (2010). Cellulosilyticum ruminicola gen. nov., sp. nov., isolated from the rumen of yak, and reclassification of Clostridium lentocellum as Cellulosilyticum lentocellum comb. nov. *International Journal of Systematic and Evolutionary Microbiology*, *60*(4), 845–849. https://doi.org/10.1099/ijs.0.014712-0

Calabrese JM, Fleming CH, Gurarie E. ctmm: an R package for analyzing animal relocation data as a continuous-time stochastic process. Freckleton R, editor. Methods Ecol Evol. 2016. 7(9):1124–32

Cao, M., Huang, J., Li, J., Qiao, Z., & Wang, G. (2017). Edaphobaculum flavum gen. nov., sp. nov., a member of family Chitinophagaceae, isolated from grassland soil. *International Journal of Systematic and Evolutionary Microbiology*, *67*(11), 4475-4481.

Chang, D. H., Rhee, M. S., Ahn, S., Bang, B. H., Oh, J. E., Lee, H. K., & Kim, B. C. (2015). Faecalibaculum rodentium gen. nov., sp. nov., isolated from the faeces of a laboratory mouse. *Antonie van Leeuwenhoek, International Journal of General and Molecular Microbiology*, *108*(6), 1309–1318. https://doi.org/10.1007/s10482-015-0583-3

Choo, Y. J., Lee, K., Song, J., & Cho, J. C. (2007). Puniceicoccus vermicola gen. nov., sp. nov., a novel marine bacterium, and description of Puniceicoccaceae fam. nov., Puniceicoccales ord. nov., Opitutaceae fam. nov., Opitutales ord. nov. and Opitutae classis nov. in the phylum 'Verrucomicrobia'. International journal of systematic and evolutionary microbiology, 57(3), 532-537.

Clavel, T., Charrier, C., Wenning, M., & Haller, D. (2013). Parvibacter caecicola gen. nov., sp. nov., a bacterium of the family Coriobacteriaceae isolated from the caecum of a mouse. *International Journal of Systematic and Evolutionary Microbiology*, *63*(PART7), 2642–2648. https://doi.org/10.1099/ijs.0.045344-0

Clavel, T., Duck, W., Charrier, C., Wenning, M., Elson, C., & Haller, D. (2010). Enterorhabdus caecimuris sp. nov., a member of the family Coriobacteriaceae isolated from a mouse model of spontaneous colitis, and emended description of the genus Enterorhabdus Clavel et al. 2009. In

International Journal of Systematic and Evolutionary Microbiology (Vol. 60, Issue 7, pp. 1527–1531). Microbiology Society. https://doi.org/10.1099/ijs.0.015016-0

Cohen, J., Powderly, W., & Opal, S. (Ed.). (2017). *Infectious Diseases* (4th ed.). Elsevier. https://doi.org/https://doi.org/10.1016/C2013-1-00044-3

Cox, L. M., Sohn, J., Tyrrell, K. L., Citron, D. M., Lawson, P. A., Pate, N. B., Iizumi, T., Perez-Perez, G. I., Goldstein, E. J. C., & Blaser, M. J. (2017). Description of two novel members of the family erysipelotrichaceae: Ileibacterium valens gen. nov., sp. nov. and Dubosiella newyorkensis, gen. nov., sp. nov., from the murine intestine, and emendation to the description of Faecalibacterium rodentium. *International Journal of Systematic and Evolutionary Microbiology*, *67*(5), 1247–1254. https://doi.org/10.1099/ijsem.0.001793

Davidov, Y., & Jurkevitch, E. (2004). Diversity and evolution of Bdellovibrio-and-like organisms (BALOs), reclassification of Bacteriovorax starrii as Peredibacter starrii gen. nov., comb. nov., and description of the Bacteriovorax–Peredibacter clade as Bacteriovoracaceae fam. nov. *International journal of systematic and evolutionary microbiology*, *54*(5), 1439-1452.

de Meyer, S. E., Coorevits, A., & Willems, A. (2012). Tardiphaga robiniae gen. nov., sp. nov., a new genus in the family Bradyrhizobiaceae isolated from Robinia pseudoacacia in Flanders (Belgium). *Systematic and Applied Microbiology*, *35*(4), 205–214. https://doi.org/10.1016/j.syapm.2012.02.002

Downes, J., Dewhirst, F. E., Tanner, A. C., & Wade, W. G. (2013). Description of Alloprevotella rava gen. nov., sp. nov., isolated from the human oral cavity, and reclassification of Prevotella tannerae Moore et al. 1994 as Alloprevotella tannerae gen. nov., comb. nov. *International journal of systematic and evolutionary microbiology*, *63*(Pt_4), 1214-1218.

Fahrbach, M., Kuever, J., Remesch, M., Huber, B. E., Kampfer, P., Dott, W., & Hollender, J. (2008). Steroidobacter denitrificans gen. nov., sp. nov., a steroidal hormone-degrading gammaproteobacterium. *International Journal of Systematic and Evolutionary Microbiology*, *58*(9), 2215-2223.

Falagán, C., & Johnson, D. B. (2014). Acidibacter ferrireducens gen. nov., sp. nov.: an acidophilic ferric iron-reducing gammaproteobacterium. *Extremophiles*, *18*(6), 1067-1073.

Fleming CH, Fagan WF, Mueller T, Olson KA, Leimgruber P, Calabrese JM, et al. Rigorous home range estimation with movement data: A new autocorrelated kernel density estimator. Ecology. 2015 1;96(5):1182–8.

Fudou, R., Jojima, Y., Iizuka, T., & Yamanaka, S. (2002). Haliangium ochraceum gen. nov., sp. nov. and Haliangium tepidum sp. nov.: novel moderately halophilic myxobacteria isolated from coastal saline environments. *The Journal of general and applied microbiology*, *48*(2), 109-115.

Garcia, R. O., Reichenbach, H., Ring, M. W., & Muller, R. (2009). Phaselicystis flava gen. nov., sp. nov., an arachidonic acid-containing soil myxobacterium, and the description of Phaselicystidaceae fam. nov. *International journal of systematic and evolutionary microbiology*, *59*(6), 1524-1530.

Geissinger, O., Herlemann, D. P. R., Morschel, E., Maier, U. G., & Brune, A. (2009). The ultramicrobacterium "Elusimicrobium minutum" gen. nov., sp. nov., the first cultivated representative of the termite group 1 phylum. *Applied and Environmental Microbiology*, *75*(9), 2831–2840. https://doi.org/10.1128/AEM.02697-08

Gerritsen, J., Fuentes, S., Grievink, W., van Niftrik, L., Tindall, B. J., Timmerman, H. M., ... & Smidt, H. (2014). Characterization of Romboutsia ilealis gen. nov., sp. nov., isolated from the gastro-intestinal tract of a rat, and proposal for the reclassification of five closely related members of the genus Clostridium into the genera Romboutsia gen. nov., Intestinibacter gen. nov., Terrisporobacter gen. nov. and Asaccharospora gen. nov. *International journal of systematic and evolutionary microbiology*, *64*(Pt_5), 1600-1616.

Gerritsen, J., Hornung, B., Ritari, J., Paulin, L., Rijkers, G., Schaap, P., de Vos, W., & Smidt, H. (2019). A comparative and functional genomics analysis of the genus Romboutsia provides insight into adaptation to an intestinal lifestyle. *BioRxiv*, 845511. https://doi.org/10.1101/845511

Gophna, U., Konikoff, T., & Nielsen, H. B. (2017). Oscillospira and related bacteria–From metagenomic species to metabolic features. *Environmental microbiology*, *19*(3), 835-841.

Greetham, H. L., Gibson, G. R., Giffard, C., Hippe, H., Merkhoffer, B., Steiner, U., ... & Collins, M. D. (2004). Allobaculum stercoricanis gen. nov., sp. nov., isolated from canine feces. Anaerobe, 10(5), 301-307.

Hahnke, R. L., Meier-Kolthoff, J. P., García-López, M., Mukherjee, S., Huntemann, M., Ivanova, N. N., ... & Göker, M. (2016). Genome-based taxonomic classification of Bacteroidetes. *Frontiers in microbiology*, *7*, 2003.

Hardham, J. M., King, K. W., Dreier, K., Wong, J., Strietzel, C., Eversole, R. R., ... & Evans, R. T. (2008). Transfer of Bacteroides splanchnicus to Odoribacter gen. nov. as Odoribacter splanchnicus comb. nov., and description of Odoribacter denticanis sp. nov., isolated from the crevicular spaces of canine periodontitis patients. International journal of systematic and evolutionary microbiology, 58(1), 103-109.Hwang, W. M., Ko, Y., Kim, J. H., & Kang, K. (2018). Ahniella affigens gen. nov., sp. nov., a gammaproteobacterium isolated from sandy soil near a stream. *International Journal of Systematic and Evolutionary Microbiology*, *68*(8), 2478-2484.

Iino, T., Mori, K., Tanaka, K., Suzuki, K. I., & Harayama, S. (2007). Oscillibacter valericigenes gen. nov., sp. nov., a valerate-producing anaerobic bacterium isolated from the alimentary canal of a Japanese corbicula clam. *International Journal of Systematic and Evolutionary Microbiology*, *57*(8), 1840–1845. https://doi.org/10.1099/ijs.0.64717-0

Jeong, H., Lim, Y. W., Yi, H., Sekiguchi, Y., Kamagata, Y., & Chun, J. (2007). Anaerosporobacter mobilis gen. nov., sp. nov., isolated from forest soil. *International Journal of Systematic and Evolutionary Microbiology*, *57*(8), 1784–1787. https://doi.org/10.1099/ijs.0.63283-0

Kanso, S., & Patel, B. K. C. (2003). Microvirga subterranea gen. nov., sp. nov., a moderate thermophile from a deep subsurface Australian thermal aquifer. *International Journal of Systematic and Evolutionary Microbiology*, *53*(2), 401–406. https://doi.org/10.1099/ijs.0.02348-0

Kämpfer, P., Young, C. C., Arun, A. B., Shen, F. T., Jäckel, U., Rosselló-Mora, R., ... & Rekha, P. D. (2006). Pseudolabrys taiwanensis gen. nov., sp. nov., an alphaproteobacterium isolated from soil. *International journal of systematic and evolutionary microbiology*, *56*(10), 2469-2472.

Kim, B. C., Jeon, B. S., Kim, S., Kim, H., Um, Y., & Sang, B. I. (2015). Caproiciproducens galactitolivorans gen. Nov., sp. nov., a bacterium capable of producing caproic acid from galactitol, isolated from a wastewater treatment plant. *International Journal of Systematic and Evolutionary Microbiology*, *65*(12), 4902–4908. https://doi.org/10.1099/ijsem.0.000665

Kim, J. J., Alkawally, M., Brady, A. L., Rijpstra, W. I. C., Sinninghe Damsté, J. S., & Dunfield, P. F. (2013). Chryseolinea serpens gen. nov., sp. nov., a member of the phylum Bacteroidetes isolated from soil. *International journal of systematic and evolutionary microbiology*, *63*(Pt_2), 654-660.

Kim, S. J., Park, J. H., Lim, J. M., Ahn, J. H., Anandham, R., Weon, H. Y., & Kwon, S. W. (2014). Parafilimonas terrae gen. nov., sp. nov., isolated from greenhouse soil. *International journal of systematic and evolutionary microbiology*, *64*(Pt_9), 3040-3045

Kläring, K., Hanske, L., Bui, N., Charrier, C., Blaut, M., Haller, D., Plugge, C. M., & Clavel, T. (2013). Intestinimonas butyriciproducens gen. nov., sp. nov., a butyrate-producing bacterium from the mouse intestine. *International Journal of Systematic and Evolutionary Microbiology*, *63*(PART 12), 4606–4612. https://doi.org/10.1099/ijs.0.051441-0

Krishnamurthi, S., Bhattacharya, A., Mayilraj, S., Saha, P., Schumann, P., & Chakrabarti, T. (2009). Description of Paenisporosarcina quisquiliarum gen. nov., sp. nov., and reclassification of Sporosarcina macmurdoensis Reddy et al. 2003 as Paenisporosarcina macmurdoensis comb. nov. *International Journal of Systematic and Evolutionary Microbiology*, *59*(6), 1364–1370. https://doi.org/10.1099/ijs.0.65130-0

Krishnamurthi, S., Chakrabarti, T., & Stackebrandt, E. (2009). Re-examination of the taxonomic position of Bacillus silvestris Rheims et al. 1999 and proposal to transfer it to Solibacillus gen. nov. as Solibacillus silvestris comb. nov. *International Journal of Systematic and Evolutionary Microbiology*, *59*(5), 1054–1058. https://doi.org/10.1099/ijs.0.65742-0

Kwon, K. K., Woo, J. H., Yang, S. H., Kang, J. H., Kang, S. G., Kim, S. J., ... & Kato, C. (2007). Altererythrobacter epoxidivorans gen. nov., sp. nov., an epoxide hydrolase-active, mesophilic marine bacterium isolated from cold-seep sediment, and reclassification of Erythrobacter

luteolus Yoon et al. 2005 as Altererythrobacter luteolus comb. nov. *International journal of systematic and evolutionary microbiology*, *57*(10), 2207-2211.

Kwon, S. W., Kim, B. Y., Weon, H. Y., Baek, Y. K., & Go, S. J. (2007). Arenimonas donghaensis gen. nov., sp. nov., isolated from seashore sand. *International journal of systematic and evolutionary microbiology*, *57*(5), 954-958.

Lagkouvardos, I., Lesker, T. R., Hitch, T. C. A., Gálvez, E. J. C., Smit, N., Neuhaus, K., Wang, J., Baines, J. F., Abt, B., Stecher, B., Overmann, J., Strowig, T., & Clavel, T. (2019). Sequence and cultivation study of Muribaculaceae reveals novel species, host preference, and functional potential of this yet undescribed family. *Microbiome*, *7*(1), 28. https://doi.org/10.1186/s40168-019-0637-2

La Scola, B., Barrassi, L., & Raoult, D. (2004). A novel alpha-Proteobacterium, Nordella oligomobilis gen. nov., sp. nov., isolated by using amoebal co-cultures. *Research in microbiology*, *155*(1), 47-51.

Lee, K. B., Liu, C. T., Anzai, Y., Kim, H., Aono, T., & Oyaizu, H. (2005). The hierarchical system of the 'Alphaproteobacteria': description of Hyphomonadaceae fam. nov., Xanthobacteraceae fam. nov. and Erythrobacteraceae fam. nov. International Journal of Systematic and Evolutionary Microbiology, 55(5), 1907-1919.

Liu, M. J., Jin, C. Z., Asem, M. D., Ju, Y. J., Park, D. J., Salam, N., ... & Kim, C. J. (2018). Aurantisolimonas haloimpatiens gen. nov., sp. nov., a bacterium isolated from soil. *International Journal of Systematic and Evolutionary Microbiology*, *68*(5), 1552-1559.

Liu, Q., Liu, H. C., Zhou, Y. G., & Xin, Y. H. (2019). Stenotrophobium rhamnosiphilum gen. nov., sp. nov., isolated from a glacier, proposal of Steroidobacteraceae fam. nov. in Nevskiales and emended description of the family Nevskiaceae. *International Journal of Systematic and Evolutionary Microbiology*, *69*(5), 1404-1410.

Lv, Y. Y., Wang, J., Chen, M. H., You, J., & Qiu, L. H. (2016). Dinghuibacter silviterrae gen. nov., sp. nov., isolated from forest soil. *International journal of systematic and evolutionary microbiology*, *66*(4), 1785-1791.

Kim, J. J., Alkawally, M., Brady, A. L., Rijpstra, W. I. C., Sinninghe Damsté, J. S., & Dunfield, P. F. (2013). Chryseolinea serpens gen. nov., sp. nov., a member of the phylum Bacteroidetes isolated from soil. *International journal of systematic and evolutionary microbiology*, *63*(Pt_2), 654-660.

Kim, S. J., Park, J. H., Lim, J. M., Ahn, J. H., Anandham, R., Weon, H. Y., & Kwon, S. W. (2014). Parafilimonas terrae gen. nov., sp. nov., isolated from greenhouse soil. *International journal of systematic and evolutionary microbiology*, *64*(Pt_9), 3040-3045

Kim, B. C., Jeon, B. S., Kim, S., Kim, H., Um, Y., & Sang, B. I. (2015). Caproiciproducens galactitolivorans gen. nov., sp. nov., a bacterium capable of producing caproic acid from galactitol, isolated from a wastewater treatment plant. *International journal of systematic and evolutionary microbiology*, *65*(Pt_12), 4902-4908.
Kwon, S. W., Kim, B. Y., Weon, H. Y., Baek, Y. K., & Go, S. J. (2007). Arenimonas donghaensis gen. nov., sp. nov., isolated from seashore sand. *International journal of systematic and evolutionary microbiology*, *57*(5), 954-958.
Mailhe, M., Ricaboni, D., Benezech, A., Cadoret, F., Fournier, P. E., & Raoult, D. (2017). 'Millionella massiliensis' gen. nov., sp. nov., a new bacterial species isolated from human right colon. *New Microbes and New Infections*, *17*, 11–12. https://doi.org/10.1016/j.nmni.2016.11.016

Mailhe, M., Ricaboni, D., Vitton, V., Cadoret, F., Fournier, P. E., & Raoult, D. (2017). 'Angelakisella massiliensis' gen. nov., sp. nov., a new bacterial species isolated from human ileum. *New Microbes and New Infections*, *16*, 51–53. https://doi.org/10.1016/j.nmni.2017.01.003

Matsumoto, A., Kasai, H., Matsuo, Y., Ōmura, S., Shizuri, Y., & Takahashi, Y. (2009). Ilumatobacter fluminis gen. nov., sp. nov., a novel actinobacterium isolated from the sediment of an estuary. *The Journal of general and applied microbiology*, *55*(3), 201-205.

Maturana, J. L., & Cárdenas, J. P. (2021). Insights on the evolutionary genomics of the Blautia genus: potential new species and genetic content among lineages. *Frontiers in Microbiology*, *12*, 660920.

Mediannikov, O., Sekeyová, Z., Birg, M.-L., & Raoult, D. (2010). A Novel Obligate Intracellular Gamma-Proteobacterium Associated with Ixodid Ticks, Diplorickettsia massiliensis, Gen. Nov., Sp. Nov. *PLoS ONE*, *5*(7), e11478. https://doi.org/10.1371/journal.pone.0011478

Minich, J. J.,Sanders, J. G., Amir, A., Humphrey, G., Gilbert, J. A., & Knight, R. (2019). Quantifying and Understanding Well-to-Well Contamination in Microbiome Research. *MSystems*, *4*(4). https://doi.org/10.1128/msystems.00186-19

Morotomi, M., Nagai, F., Sakon, H., & Tanaka, R. (2009). Paraprevotella clara gen. nov., sp. nov. and Paraprevotella xylaniphila sp. nov., members of the family 'Prevotellaceae'isolated from human faeces. *International journal of systematic and evolutionary microbiology*, *59*(8), 1895-1900.

Morotomi, M., Nagai, F., & Watanabe, Y. (2011). Parasutterella secunda sp. nov., isolated from human faeces and proposal of Sutterellaceae fam. nov. in the order Burkholderiales. *International journal of systematic and evolutionary microbiology*, *61*(3), 637-643.

Morotomi, M., Nagai, F., & Watanabe, Y. (2012). Description of Christensenella minuta gen. nov., sp. nov., isolated from human faeces, which forms a distinct branch in the order Clostridiales, and proposal of Christensenellaceae fam. nov. International journal of systematic and evolutionary microbiology, 62(1), 144-149.

Nagai, F., Morotomi, M., Sakon, H., & Tanaka, R. (2009). Parasutterella excrementihominis gen. nov., sp. nov., a member of the family Alcaligenaceae isolated from human faeces. *International Journal of Systematic and Evolutionary Microbiology*, *59*(7), 1793–1797. https://doi.org/10.1099/ijs.0.002519-0

Nakai, R., Nishijima, M., Tazato, N., Handa, Y., Karray, F., Sayadi, S., ... & Naganuma, T. (2014). Oligoflexus tunisiensis gen. nov., sp. nov., a Gram-negative, aerobic, filamentous bacterium of a novel proteobacterial lineage, and description of Oligoflexaceae fam. nov., Oligoflexales ord. nov. and Oligoflexia classis nov. *International journal of systematic and evolutionary microbiology*, *64*(Pt 10), 3353.

Ormerod, K. L., Wood, D. L. A., Lachner, N., Gellatly, S. L., Daly, J. N., Parsons, J. D., Dal'Molin, C. G. O., Palfreyman, R. W., Nielsen, L. K., Cooper, M. A., Morrison, M., Hansbro, P. M., & Hugenholtz, P. (2016). Genomic characterization of the uncultured Bacteroidales family S24-7 inhabiting the guts of homeothermic animals. *Microbiome*, *4*(1), 36. https://doi.org/10.1186/s40168-016-0181-2

Pagnier, I., Raoult, D., & La Scola, B. (2011). Isolation and characterization of Reyranella massiliensis gen. nov., sp. nov. from freshwater samples by using an amoeba co-culture procedure. *International Journal of Systematic and Evolutionary Microbiology*, *61*(9), 2151-2154.

Pfeiffer, N., Desmarchelier, C., Blaut, M., Daniel, H., Haller, D., & Clavel, T. (2012). Acetatifactor muris gen. nov., sp. nov., a novel bacterium isolated from the intestine of an obese mouse. *Archives of Microbiology*, *194*(11), 901–907. https://doi.org/10.1007/s00203-012-0822-1

Powell RA, Mitchell MS. What is a home range? J Mammal. 2012. 93(4):948–58.

Prosser, J. I., Head, I. M., & Stein, L. Y. (2014). The family Nitrosomonadaceae. In *The Prokaryotes: Alphaproteobacteria and Betaproteobacteria* (Vol. 9783642301971, pp. 901–918). Springer-Verlag Berlin Heidelberg. https://doi.org/10.1007/978-3-642-30197-1_372

Rivas, R., García-Fraile, P., Zurdo-Piñeiro, J. L., Mateos, P. F., Martínez-Molina, E., Bedmar, E. J., Sánchez-Raya, J., & Velázquez, E. (2008). Saccharibacillus sacchari gen. nov., sp. nov., isolated from sugar cane. *International Journal of Systematic and Evolutionary Microbiology*, *58*(8), 1850–1854. https://doi.org/10.1099/ijs.0.65499-0

Sakamoto, M., & Benno, Y. (2006). Reclassification of Bacteroides distasonis, Bacteroides goldsteinii and Bacteroides merdae as Parabacteroides distasonis gen. nov., comb. nov., Parabacteroides goldsteinii comb. nov. and Parabacteroides merdae comb. nov. *International journal of systematic and evolutionary microbiology*, *56*(7), 1599-1605.

Schnupf, P., Gaboriau-Routhiau, V., Gros, M., Friedman, R., Moya-Nilges, M., Nigro, G., Cerf-Bensussan, N., & Sansonetti, P. J. (2015). Growth and host interaction of mouse segmented filamentous bacteria in vitro. *Nature*, *520*(7545), 99–103. https://doi.org/10.1038/nature14027

Shelobolina, E. S., Nevin, K. P., Blakeney-Hayward, J. D., Johnsen, C. v., Plaia, T. W., Krader, P., Woodard, T., Holmes, D. E., VanPraagh, C. G., & Lovley, D. R. (2007). Geobacter pickeringii sp. nov., Geobacter argillaceus sp. nov. and Pelosinus fermentans gen. nov., sp. nov., isolated from subsurface kaolin lenses. *International Journal of Systematic and Evolutionary Microbiology*, *57*(1), 126–135. https://doi.org/10.1099/ijs.0.64221-0

Suzuki, T. A., Martins, F. M., & Nachman, M. W. (2019). Altitudinal variation of the gut microbiota in wild house mice. *Molecular Ecology*, *28*(9), 2378–2390. https://doi.org/10.1111/mec.14905

Stackebrandt, E., Verbarg, S., Frühling, A., Busse, H. J., & Tindall, B. J. (2009). Dissection of the genus Methylibium: reclassification of Methylibium fulvum as Rhizobacter fulvus comb. nov., Methylibium aquaticum as Piscinibacter aquaticus gen. nov., comb. nov. and Methylibium subsaxonicum as Rivibacter subsaxonicus gen. nov., comb. nov. and emended descriptions of the genera Rhizobacter and Methylibium. *International journal of systematic and evolutionary microbiology*, *59*(10), 2552-2560.

Takeuchi, M., Hamana, K., & Hiraishi, A. (2001). Proposal of the genus Sphingomonas sensu stricto and three new genera, Sphingobium, Novosphingobium and Sphingopyxis, on the basis of phylogenetic and chemotaxonomic analyses. *International Journal of Systematic and Evolutionary Microbiology*, *51*(4), 1405–1417. https://doi.org/10.1099/00207713-51-4-1405.

Trachsel, J., Humphrey, S., & Allen, H. K. (2018). Butyricicoccus porcorum sp. nov., a butyrate-producing bacterium from swine intestinal tract. *International Journal of Systematic and Evolutionary Microbiology*, *68*(5), 1737–1742. https://doi.org/10.1099/ijsem.0.002738

Tindall, B. J. (2019). The names Hungateiclostridium Zhang et al. 2018, Hungateiclostridium thermocellum (Viljoen et al. 1926) Zhang et al. 2018, Hungateiclostridium cellulolyticum (Patel et al. 1980) Zhang et al. 2018, Hungateiclostridium aldrichii (Yang et al. 1990) Zhang et al. 2018, Hungateiclostridium alkalicellulosi (Zhilina et al. 2006) Zhang et al. 2018, Hungateiclostridium clariflavum (Shiratori et al. 2009) Zhang et al. 2018, Hungateiclostridium straminisolvens (Kato et al. 2004) Zhang et al. 2018 and .... *International Journal of Systematic and Evolutionary Microbiology*, *69*(12), 3927-3932.

Tirandaz, H., Dastgheib, S. M. M., Amoozegar, M. A., Shavandi, M., de la Haba, R. R., & Ventosa, A. (2015). Pseudorhodoplanes sinuspersici gen. nov., sp. nov., isolated from oil-contaminated soil. *International Journal of Systematic and Evolutionary Microbiology*, *65*(Pt_12), 4743-4748.

Weon, H. Y., Kim, B. Y., Yoo, S. H., Lee, S. Y., Kwon, S. W., Go, S. J., & Stackebrandt, E. (2006). Niastella koreensis gen. nov., sp. nov. and Niastella yeongjuensis sp. nov., novel members of the phylum Bacteroidetes, isolated from soil cultivated with Korean ginseng. *International Journal of Systematic and Evolutionary Microbiology*, *56*(8), 1777-1782.

Weon, H. Y., Kim, B. Y., Lee, C. M., Hong, S. B., Jeon, Y. A., Koo, B. S., & Kwon, S. W. (2009). Solitalea koreensis gen. nov., sp. nov. and the reclassification of [Flexibacter] canadensis as Solitalea canadensis comb. nov. *International journal of systematic and evolutionary microbiology*, *59*(8), 1969-1975.

Yoon, J. H., Kang, S. J., Lee, S. Y., Lee, J. S., & Park, S. (2011). Ohtaekwangia koreensis gen. nov., sp. nov. and Ohtaekwangia kribbensis sp. nov., isolated from marine sand, deep-branching members of the phylum Bacteroidetes. *International journal of systematic and evolutionary microbiology*, *61*(5), 1066-1072.

Yutin, N., & Galperin, M. Y. (2013). A genomic update on clostridial phylogeny: Gram-negative spore formers and other misplaced clostridia. *Environmental Microbiology*, *15*(10), 2631–2641. https://doi.org/10.1111/1462-2920.12173

Zgurskaya, H. I., Evtushenko, L. I., Akimov, V. N., & Kalakoutskii, L. V. (1993). Rathayibacter gen. nov., including the species Rathayibacter rathayi comb. nov., Rathayibacter tritici comb. nov., Rathayibacter iranicus comb. nov., and six strains from annual grasses. International Journal of Systematic and Evolutionary Microbiology, 43(1), 143-149.

Zhang, K., Tang, Y., Zhang, L., Dai, J., Wang, Y., Luo, X., ... & Fang, C. (2009). Parasegetibacter luojiensis gen. nov., sp. nov., a member of the phylum Bacteroidetes isolated from a forest soil. *International journal of systematic and evolutionary microbiology*, *59*(12), 3058-3062.
