# Supplementary material for: Social and environmental transmission spread different sets of gut microbes in wild mice
Source: Nat Ecol Evol. 2024 May 1;8(5):972–85. doi: 10.1038/s41559-024-02381-0 (PMC11090834; doi:10.1038/s41559-024-02381-0)
Supplement: Supplementary file 2 — Reporting Summary [file 41559_2024_2381_MOESM2_ESM.pdf]

nature portfolio

Corresponding author(s): Aura Raulo
Sarah Knowles

Last updated by author(s): 18th of August 2023

Reporting Summary

Nature Portfolio wishes to improve the reproducibility of the work that we publish. This form provides structure for consistency and transparency in reporting. For further information on Nature Portfolio policies, see our Editorial Policies and the Editorial Policy Checklist.

Statistics

For all statistical analyses, confirm that the following items are present in the figure legend, table legend, main text, or Methods section.

n/a	Confirmed	
☐	☒	The exact sample size (*n*) for each experimental group/condition, given as a discrete number and unit of measurement
☐	☒	A statement on whether measurements were taken from distinct samples or whether the same sample was measured repeatedly
☐	☒	The statistical test(s) used AND whether they are one- or two-sided<br>*Only common tests should be described solely by name; describe more complex techniques in the Methods section.*
☐	☒	A description of all covariates tested
☐	☒	A description of any assumptions or corrections, such as tests of normality and adjustment for multiple comparisons
☐	☒	A full description of the statistical parameters including central tendency (e.g. means) or other basic estimates (e.g. regression coefficient) AND variation (e.g. standard deviation) or associated estimates of uncertainty (e.g. confidence intervals)
☒	☐	For null hypothesis testing, the test statistic (e.g. *F*, *t*, *r*) with confidence intervals, effect sizes, degrees of freedom and *P* value noted<br>*Give P values as exact values whenever suitable.*
☐	☒	For Bayesian analysis, information on the choice of priors and Markov chain Monte Carlo settings
☒	☐	For hierarchical and complex designs, identification of the appropriate level for tests and full reporting of outcomes
☐	☒	Estimates of effect sizes (e.g. Cohen's *d*, Pearson's *r*), indicating how they were calculated

Our web collection on statistics for biologists contains articles on many of the points above.

Software and code

Policy information about availability of computer code

Data collection	No software was used to collect the data in this study.
Data analysis	- Social network analysis was conducted using a custom method available in author's GitHub page: https://github.com/nuorenarra/Social-Network-Analysis<br>- Main statistical analyses were done using custom designed dyadic glmm models utilising brms package in R, and the drop model approach for calculating importance scores for each bacterial genus for each signal of interest were done using custom code. Both of these are available at the author's GitHub page: https://github.com/nuorenarra/Analysing-dyadic-data-with-brms

For manuscripts utilizing custom algorithms or software that are central to the research but not yet described in published literature, software must be made available to editors and reviewers. We strongly encourage code deposition in a community repository (e.g. GitHub). See the Nature Portfolio guidelines for submitting code & software for further information.

Data

Policy information about availability of data

All manuscripts must include a data availability statement. This statement should provide the following information, where applicable:

- Accession codes, unique identifiers, or web links for publicly available datasets
- A description of any restrictions on data availability
- For clinical datasets or third party data, please ensure that the statement adheres to our policy

All data used in this study is publically available through Environmental Information Data Centre (DOI: https://doi.org/10.5285/043513e5-406c-4477-89aa-c96059acb232).

Research involving human participants, their data, or biological material

Policy information about studies with human participants or human data. See also policy information about sex, gender (identity/presentation), and sexual orientation and race, ethnicity and racism.

Reporting on sex and gender	N/A
Reporting on race, ethnicity, or other socially relevant groupings	N/A
Population characteristics	N/A
Recruitment	N/A
Ethics oversight	This work was carried out under the UK Home Office project license ASPA (Animals (Scientific Procedures) Act), Licence number PB0178858 held by SCLK

Note that full information on the approval of the study protocol must also be provided in the manuscript.

Field-specific reporting

Please select the one below that is the best fit for your research. If you are not sure, read the appropriate sections before making your selection.

☐ Life sciences ☐ Behavioural & social sciences ☒ Ecological, evolutionary & environmental sciences

For a reference copy of the document with all sections, see nature.com/documents/nr-reporting-summary-flat.pdf

Ecological, evolutionary & environmental sciences study design

All studies must disclose on these points even when the disclosure is negative.

Study description	192 wild mood mice were trapped and implanted with a subcutaneous identification tag and subsequently their movements were tracked with custom made loggers for 10 months. 157 mice were successfully tracked. From 133 of these mice a faecal sample was collected from which their gut microbial profile was analysed by sequencing the 16S rRNA marker gene. The tracking data together with data on microhabitat variation in the study area were used to derive social networks, home range overlap measures and microhabitat similarity estimates among the mice. These data were used to statistically model the drives of gut microbiome variation to explore how gut microbes transmit between individual mice. Specifically, we used a dyadic Bayesian glmm model with data containing all pairwise comparisons of the mice, yo predict the similarity of their gut microbiome with their association in the social network, their home range overlap and microhabitat similarity among other covariates.
Research sample	The subjects of this study were 192 mice trapped and tagged on a 4 ha study area in Holly Hill, Wytham Woods, Oxford, UK between November 2018 and November 2019. Mice were trapped opportunistically, and the data included mice of different age groups (starting from independently moving juveniles) and sexes. Based on recapture rates, the tagged mice represented a great majority of the mice present in the area (>85% of the population).
Sampling strategy	Mice were trapped opportunistically within the study area, and the sample size was thus defined by the size of the study area. We chose 4 ha area based on previous knowledge on seasonally shifting population densities, to ensure we would have a statistically convincing number of individuals (~30) even in the low-population density time of the year (Spring)
Data collection	Field data collection involved fortnightly trapping to tag mice and collect samples, alongside continuous passive tracking of tagged individuals using RFID technology. The field data collection was led by Aura Raulo, with the help of Sarah Knowles, Jarrah Dale, Curt Lamberth, Holly English and Eveliina Hanski. The focus study area, where mouse behavior was tracked, was a 2.56 ha (160m x 160m) "core grid", but to minimise edge effects mice were trapped and tagged from an area larger than this core grid, from a 4 ha (200m x 200m) "extended grid" spanning up to 40 meters outside of the core. Trapping sessions were carried out in the area from November 2018 to November 2019, with captured mice aged and sexed, and injected with a subcutaneous PIT-tag for permanent identification

and tracking. After processing, all individuals were immediately released at the exact location they were trapped. Faecal samples for microbiota analysis were collected from the traps of identified individuals into sterile sample tubes with sterile tweezers and frozen at -80oC within 4 hours of collection. All traps showing signs of rodent presence were carefully washed and sterilised in bleach solution before the next trapping session, to eliminate cross-contamination. Additionally, in the beginning of the study period (between November 2018-February 2019), 25 soil samples were collected from around the 4 ha extended grid to serve as a general reference to the local soil microbiota. A soil sample was collected by digging a spoonful of soil (~200 mg) from 3 cm underground, creating a mix from three digging spots within a meter of a mouse trapping location.
Mouse behaviour was monitored with a set of 60 custom-built RFID-loggers distributed across the study site, recording the time-stamped presence of any individual that came within its read range (~1m2). Loggers were unbaited, positioned evenly across the grid and rotated fortnightly to ensure even spatial coverage of the area. This scheme meant that each 10 x 10 m grid cell of the study site was covered by a logger for a fortnight every two months, i.e., 25% of the time. In addition to these evenly spaced “above-ground” loggers used to derive social networks, to derive home range estimates we also included data from an extra set of 60 loggers positioned at entrances to mouse burrows between July-November. These “burrow loggers” were not rotated but were distributed approximately evenly across the study area.
Shortly after the study (May, 2020), we completed a thorough survey of vegetation and microhabitat variation across the study site, in which the percentage cover by each of the eight main ground cover types in the area was recorded for each 10 x 10m grid cell of the plot.

Timing and spatial scale

Mice were trapped fortnightly on a 4 ha study area in Holly Hill, Wytham Woods, Oxford, UK between November 2018 and November 2019.

Data exclusions

For the statistical analyses linking tracking data and microbiota data, we excluded tracking data from mice who were not successfulky profiled for microbiome. 192 mice were trapped in total, of which 157 were successfully tracked with the loggers within the study area and 133 of these had a succesfully profiled microbiome and 104 of these also had enough tracking data for home range construction.

In total 241 samples from 104 of the 192 tagged mice were used in the statistical models.

From the microbiome data we dropped samples with read count lower than required for reliable diversity estimation (6000 reads for gut microbiota samples, 800 for soil microbiota samples). We further excluded sequences that were present in the data as singletons or doubletons (observed <3 times across the data). We also excluded all sequences assigned to Cyanobacteria or Mitichondria as they are not gut microbes.

For the models linking bacterial genus phenotypes with their transmission importance scores, we used 188 (out of 234) genera, for which we could reliably find information on both their aerotolerance and spore-formation abilities, and which were not known to be mixed in these phenotypes within a genus.

Reproducibility

As part of this study we reproduce the main findings of another similar study using another wood mouse population in another County (Raulo et al., 2021, ISMEJ). We have documented our methods clearly enough that the whole study could be reproduced in another population with the same exact methods. All lab protocols are public and all code for constructing the social networks and running the statistical analyses is published in GitHub

Randomization

The microbiota samples were randomised across DNA extraction plates and re-ordered again for pcr plates in Library preparation step to avoid confounding sampling time effect with batch effects. Despite this a slight temporal pattern remains: The first sequencing batch only contains samples from the first half of the study period Feb-July while the second sequencing batch contains samples from across the whole study period Feb-Nov

Blinding

This was an observational data collection study using wild individuals, so blinding was not applicable.

Did the study involve field work? ☒ Yes ☐ No

Field work, collection and transport

Field conditions

The study area was in a temperate woodland in England, temperatures ranging from +25 to -5 degrees celsius depending on the time of the year. When temperatures were below 3 degrees Celsius or below below 6 and rainy, now trapping was carried out for welfare reasons.

Location

Holly Hill in Wytham Woods, Oxford, UK (51.77 °N, -1.33°S).

Access & import/export

Animals were trapped and sampled under Project License (PPL) PB0178858 granted to Sarah Knowles or this work, as well as ethical approval of Oxford University Animal Welfare and Ethical Review Board. In addition all field workers handling animals were properly trained and held personal licenses (PIL) for work with wild rodents.

Disturbance

Mild stress caused for the mice upon repeated trapping and releasing them was mitigated by a well-established routine which minimised moving on the study area and made our actions more predictable for the animal study subjects. Mice were given peanuts as part of the trapping as a lure/compensation. We also caused some minor trampling of ground vegetation.

Reporting for specific materials, systems and methods

We require information from authors about some types of materials, experimental systems and methods used in many studies. Here, indicate whether each material, system or method listed is relevant to your study. If you are not sure if a list item applies to your research, read the appropriate section before selecting a response.

Materials & experimental systems

n/a	Involved in the study
☒	☐ Antibodies
☒	☐ Eukaryotic cell lines
☒	☐ Palaeontology and archaeology
☐	☒ Animals and other organisms
☒	☐ Clinical data
☒	☐ Dual use research of concern
☒	☐ Plants

Methods

n/a	Involved in the study
☒	☐ ChIP-seq
☒	☐ Flow cytometry
☒	☐ MRI-based neuroimaging

Animals and other research organisms

Policy information about studies involving animals; ARRIVE guidelines recommended for reporting animal research, and Sex and Gender in Research

Laboratory animals	Study involved no laboratory animals
Wild animals	Wild rodents were trapped fortnightly with Sherman traps. Traps were set at dusk and collected at dawn. All trapped individuals were first checked for welfare concerns and prioritised accordingly. Four species of rodents were trapped: Wood mice (Apodemus sylvaticus, great majority of trapped individuals), yellow-necked mice (Apodemus flavicollis), bank vole (Myodes glaerolus) and common shrew (Sorex araneous, very rarely). All trapped rodents (all captured animals except shrews) were tagged and data on their age and sex was collected. After this, trapped animals were released at their exact point of capture. Animals were released in batches alongside processing them.
Reporting on sex	Wood mice were sexed upon trapping based on anogenital distance. This is a common and reliable method for sexing rodents. Our data was not biased in terms of sex ratio (97 females, 92 males) and sex was taken in account as a covariate in all our models. Furthermore the we explored how the main effects in our data differed between different sex combinations of individuals.
Field-collected samples	Faecal samples collected from the field were frozen in -80 degrees Celsius within hours of collection and thawed for DNA extraction later.
Ethics oversight	Ethical approval was granted and monitored by Oxford University Animal Welfare and Ethical Review Boards (AWERB) based on ethical approval granted by Home Office through Project License PB0178858 . All researchers carrying out procedures on animals had a valid Personal License to do so, under this Project License.

Note that full information on the approval of the study protocol must also be provided in the manuscript.
